# Assessment of genetic diversity in *Sehima nervosum* for yield, nutritional traits and ensiling quality

**DOI:** 10.1016/j.heliyon.2025.e42033

**Published:** 2025-01-17

**Authors:** Sultan Singh, Tejveer Singh, Neeraj Kumar, Pushpendra Koli, Madan Mohan Das, Sanat Kumar Mahanta, Krishna Kumar Singh, Prakash K. Jha, PV Vara Prasad, Manoj Kumar Srivastava, Rohit Katiyar

**Affiliations:** a ICAR-Indian Grassland and Fodder Research Institute, Jhansi, 284003, Uttar Pradesh, India; b ICAR-Indian Agricultural Research Institute, New Delhi, 110012, India; c Department of Plant and Soil Sciences, Mississippi State University, Starkville, MS, 39762, USA; d Feed the Future Sustainable Intensification Innovation Lab and Department of Agronomy, Kansas State University, Manhattan, KS, 66506, USA

**Keywords:** Energy value, Germplasm, methane, *Sehima nervosum*, silage, Soluble sugar

## Abstract

The low sugar content of tropical range grasses makes them difficult to ensiling. This study identified sugar rich (>7 % on dry matter basis) accessions of *Sehima nervosum* (*SN*), which is adequate to initiate lactic acid production during ensiling. *Sehima is* a prominent range grass of *Sehima-Dichatnthium* grasslands in India followed by Africa, Pakistan, China and Australia. In this study, 92 diverse *SN* germplasm accessions were evaluated for biomass yield, nutritional quality and sugar content. Green forage yield was higher in rainy season (ranged 2.2 to 58.1 t ha^−1^) compared to post rainy season (varied between 0.48 and 44.0 t ha^−1^). The approximates values expressed (%), crude protein ranged from 3.15 to 6.10, neutral detergent fiber from 67.13 to 81.95, acid detergent fiber from 42.03 to 56.05, cellulose from 24.49 to 37.98 and lignin from 3.78 to 7.97. Sugar content varied from 27.04 to 123.32 mg g^−1^ DM. To advance this experiment, the next phase involved selecting 19 accessions of SN with >7 % sugar content on DM and promising forage yield. These accessions again evaluated for nutritional quality, subjected to in vitro tests, and then assessed for ensiling potential. Among 19 accessions, carbohydrate bound to lignin (*Cc*) ranged from 15.35 % to 25.63 % total carbohydrates. The total digestible nutrients (TDN) ranged from 33.07 % to 44.32 %, digestible energy (DE) from 1.46 to 1.95 Kcal per kg of dry matter (DM), and metabolizable energy (ME) from 1.20 to 1.60 Kcal per kg of DM. The intake of dry matter, digestible dry matter, and relative feed value varied from 1.55 to 1.77, 45.88 %–51.51 %, and 57.96 %–66.94 %, respectively. Methane production, as a percentage of the total gas, was at its lowest for IG-99-195 (7.91 %) and reached its highest for IG-01-391 (14.97 %). Silage pH ranged from 4.57 (IG-02-703) to 5.61 (IG-99-181) and lactic acid from 0.094 % to 1.774 % DM. Accessions IG-02-703, IG-02-713, and IG-2045-1 had good silage quality, with suitable pH and lactic acid levels. Planting these in pastures and grazing lands could improve the availability of quality fodder during lean periods.

## 1 Introduction

Grasslands and fodder resources are facing considerable strain, as cultivated fodder accounts for just 4 % of India's arable land, resulting in a 35.6 % shortfall in green fodder [1]. Grassland occupies about 3.5 billion hectares, distributed over 26 % of global land, 70 % of farming area, and 20 % of carbon stock globally [2]. Grasses have large genetic base with 620 genera and 10 000 species. Grass diversity is rich in the Indian sub-continent with 245 genera and 1256 species, of which one-third have fodder value [3]. Nearly 48 % of forage biomass fed to livestock globally comes from grasses [4]. Livestock grazing contributes substantially to the Indian rural economy as over 50 % of livestock depends on varying degrees of grazing in community lands, forests, and pastures. According to a survey [5], the national area of permanent pasture and grazing land (PP & GL) decreased from 120 to 102 lakh hectares between 1980–81 and 2007–08. Notable reductions in PP & GL area were seen across several states, including Karnataka, Andhra Pradesh, Maharashtra and Madhya Pradesh. Grasses yield and nutritional quality differ with species, season, growth stage, and agronomic practices. The nutrition quality of forage plays a crucial role in livestock productivity, as it relies on the availability and quantity of nutrients present in the forage [6]. Generally tropical grasses utilize as green, and hay compared to the silage. Ensiling tropical grasses face challenges like less dry matter, inadequate amount of water-soluble carbohydrate (WSC), and less energy contents [[7], [8], [9]], resulting reduces the fermentation process and subsequently decreases the chances for the adoption of tropical grasses for silage technology [10]. A study suggests that fresh grasses should have at least 3.7 % WSC or approximately 1.5 % WSC on a dry weight basis to produce superior quality silage without any additives [11]. For successful fermentation during ensiling, the WSC should be between 5 and 8.6 % DM or 2.5–3 % fresh, depending on forage DM at ensiling [12]. Furthermore, individual species and their growth stages also play important roles as pre-ensiling factors in silage quality [13]*.* In Australia, the DM content of forage used for silage-making varied between 35 and 50 % for both chopped and baled silages [14]. Tropical grasses usually low in water-soluble carbohydrate than that their counterpart temperate grasses due to which substantial variability exists in their silage quality [15]. Further tropical grasses had low lactic acid bacteria population which makes them difficult to ensile without any additives [16].

Among *Sehima* species (*S. ischaemoides, S. sulcatum, S. notatum* and *S. nervosum*) found in the Indian subcontinent, *S. nervous* is the dominant species which is also known as rat's tail grass or white grass [17]. *Sehima nervosum* (*SN)* grows well in eroded, red gravelly or stony to medium sandy, loamy soils under hot and dry climate in rainfall regions of 250–1500 mm [18]. *S. nervosum* is a valuable fodder crop due to its high nutritional content, but farmers face challenges such as limited land availability, water scarcity, and the need for improved crop varieties. Sehima–Dichanthium grasslands, one of India's five major grassland types, are predominantly found south of the Tropic of Cancer in India. These grasslands, dominated by Sehima grass, also extend to Southeast Asia, Eastern Africa, and Australia. In India, they are situated south of the Northern Great Plains, between longitudes 68° and 87°E and latitudes 8° and 24°N. It is utilized mainly as a forage resource for feeding both as stall fed and grazing ruminants and controlling soil erosion. As green fodder, it has high palatability [19] and is suitable for grazing and hay making [20]. The success of any crop improvement program lies in availability and screening genetic diversity for interested trait. The twin important aspects of forage nutrition/feeding to achieve improved animal production are its yield and the nutritional value. The ICAR-Indian Grassland and Fodder Research Institute (IGFRI) keep maintenance of SN accessions found globally [21]. Bundel Sen Ghas-1 variety developed through the selection of available germplasm, primarily aimed at achieving higher biomass [22]. However, research efforts have not yet focused on developing new varieties specifically for ensiling properties. Grasses are common and predominant in tropical and subtropical grasslands. During the rainy season, they are available in surplus as fodder, but in the lean period, farmers face a scarcity of fodder. Silage could be an excellent alternative for conserving fodder. However, grasses generally have a low sugar content, making them difficult to utilize in ensiling.

In this study, addressing the above research gap, we identified sugar-rich accessions of *Sehima* out of 92 accessions maintained at ICAR-IGFRI, Jhansi, and finalized 19 accessions for ensiling purposes, each containing more than 70 mg g⁻^1^ DM sugar content. The sugar-rich accessions were studied for nutritional parameters and silage quality indices. The study aims to identify accessions superior in sugar content and with good ensiling potential for their establishment and propagation in common grazing lands, forest areas, grasslands, pastures, and *Gaushalas* (cattle shelters). This will help bridge the forage deficit and enhance the availability of quality forage as silage during scarcity periods to sustain livestock productivity.

## 2 Materials and methods

### 2.1 Site of the experiment and germplasm maintenance

This study was done in the Research Farm of the Indian Grassland and Fodder Research Institute, Jhansi, India (25°31′ N, 78°32’ E; 237 masl). This location has a hot, semi-humid climate, with summer average temperatures of 44.5 °C, while temperature reaches to 2 °C during the winter. The soil is deep, fine loamy, and moderately well drained. During sowing, fertilizers were applied at 80 kg nitrogen, 60 kg potassium per hectare and around 30 t ha^−1^ farmyard manure. Seeds of 92 *SN* accessions were initially grown in poly bags. When seedlings attainted the height of approximately 30 cm, then during the rainy season they transplanted into the field with maintaining three checks (cv. BS-1, IG02-713, and IG-99-188) under the augmented randomized complete block design in seven blocks. Each accession was transplanted with 2 rows of plants per plot of 1 m × 3 m size.

From the *Sehima* germplasm (92 accessions), 19 accessions which exhibited sugar content more than 70 mg per gram of DM transplanted using a RCBD (3 replicates) in the rainy season. Each accession was planted in 3 m × 2.5 m plots, with 5 rows of plants per plot. The spacing was maintained at 0.50 m × 0.50 m between lines and plants, with 1.0 m between plots.

### 2.2 Collection of samples & preparation

**Fig. 1:**
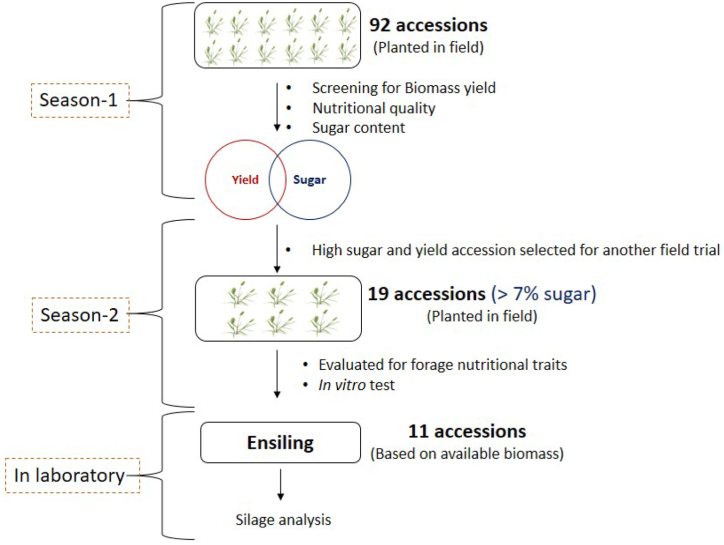
Flowchart of experiment conducted in this study.

The *Sehima* germplasm and *SN* accessions were collected from each row at the flower initiation stage in post-rainy season with mean temperature and humidity 28 °C and 77 %, respectively for nutritional evaluation. Two lots were prepared: one portion used to determine dry matter (dried at 100 °C for 72 h), while the other portion for the biochemical estimations was dried at 60 °C for same period using the VDLUFA (Verband Deutscher Landwirtschaftlicher Untersuchungs-und Forschungsanstalten) method [23]. Samples were grounded using Willey mill through a 1-mm sieve and then placed in the plastic containers for subsequent nutritional and in vitro analyses. Fresh forage yield was promptly measured post-harvest using a portable balance. A brief understanding of this study is explained in a flowchart (Fig. 1). Additionally, the 1st cut was collected during post-rainy season whereas the 2nd cut during spring season.

### 2.3 Chemical analyses

#### 2.3.1 Chemical compositions

The dry matter content, ash, nitrogen, and ether extract were determined using the Association of Official Analytical Chemists (AOAC) Method [24]. To estimate crude protein, the obtained values of nitrogen were multiplied by 6.25. Fiber fractions, including ADF, NDF, cellulose, and lignin (sa), were determined with help of a fibre apparatus (Fibra Plus FES 6, Pelican, Chennai, India) as described in an earlier method [25]. NDF was determined without the use of heat stable α-amylase and sodium sulphite. The difference between NDF and ADF was calculated as hemicellulose.

#### 2.3.2 Sugar content estimation

A 100 mg sample of ground powder, sieved to 1 mm, exposed to 80 % ethanol-water (10 mL) in a water bath at 80 °C for 30 min, followed by centrifugation at 10000 rpm for 10 min. Using the anthrone method with glucose as the standard, the sugar content was estimated [26]. The resulting blue colour at 630 nm using a UV Spectrophotometer (LABINDIA3000).

#### 2.3.3 Determination of carbohydrate fractions

Carbohydrate fractions were determined using the Cornell net carbohydrate and protein (CNCP) system [27]. This system categorizes carbohydrates into four fractions based on their degradation rates: C_A_ (rapidly degrades sugars; C_B1_ immediately degrades starch and pectin), C_B2_ (slowly degrades cell walls), and C_C_ (unavailable/lignin-bound cell walls). Total carbohydrate (tCHO) content was calculated by subtracting CP, EE and ash contents from 100. Structural carbohydrates (S_C_) were calculated by subtracting the neutral detergent insoluble protein (NDIP) from NDF, and for non-structural carbohydrate (NSC) were determined by subtracting SC from tCHO [28]. For starch estimation, samples were extracted in 80 % ethyl alcohol to solubilize free sugars, lipids, pigments, and waxes. The starch-rich residue was then solubilized with perchloric acid, and the extract was treated with anthrone-H_2_SO_4_ to determine glucose colorimetrically using glucose as the standard [26].

#### 2.3.4 Determination of protein fractions

For the estimating CP fractions in accessions, a modified CNCP system procedure [29] was employed. The protein fractions are as follows: P_A_ (non-protein nitrogen) is estimated by subtracting true CP nitrogen, precipitated with 0.3 M sodium tungstate and 0.5 M sulphuric acid, from total nitrogen; P_B1_ (buffer soluble protein) is calculated as the difference between true protein and buffer-insoluble protein, using borate phosphate buffer (pH 6.7–6.8) and freshly prepared 0.10 sodium azide solution [30]. Fraction P_B2_ (neutral detergent soluble protein) is determined by subtracting NDIP from buffer-insoluble protein, and P_B3_ (acid detergent soluble CP) is calculated as the difference of NDIP and acid detergent insoluble CP. The P_C_ fraction is considered as non-digestible.

#### 2.3.5 Determination of energy and digestible nutrients

The intake of dry matter (DMI), relative feed value (RFV), digestible dry matter (DDM), energy for lactation (NE_L_), total digestible nutrients (TDN), gain (NE_G_) and maintenance (NE_M_) were measured with help of the following equations (DMI = 120/NDF; DDM = 88.9-0.779∗ADF; RFV =(DDM∗DMI)∗0.775; TDN = 104.97-(1.302∗ADF); NE_L_ =(TDN∗0.0245)-0.012; NE_G_= (TDN∗0.029)-1.01; NE_M_ = (TDN∗0.029)-0.29) of previous equation [31]. Digestible energy (DE, KJ g^−1^ DM; DE = TDN∗0.04409) & metabolizable energy (ME, KJ g^−1^ DM) estimated by the equations given by Fonnesbeck et al., 1984 and Khalil et al., 1986 [32,33], respectively. Whereas, metabolizable energy calculated as DE x 0.821. The following equations were used to calculate digestible nutrients [34].

DCP (kg/kg DM) = (0.93∗CP%DM-3)

100

dCHO (kg/kg DM) = (digestible Organic Matter) ∗ (100-ash% Dry Matter)

100 100

dEE (kg/kg DM) = (0.96∗EE%DM-1)

100

### 2.4 In vitro study

#### 2.4.1 Inoculums preparation and the donor animals

To collect rumen liquor, four adult male sheep (*Jalauni* breed) averaging 34.3 ± 0.32 kg in body weight, housed at the Institute's Small Ruminant Experimental Farm, were exclusively fed a diet of berseem hay. The rumen liquor was collected from individual sheep before feeding, using a perforated tube and a vacuum pressure pump, and stored in a in a pre-warmed thermos. The rumen liquor collected from each animal was pooled together, passed through several layers of muslin cloth, kept in a water bath at 39 °C, and infused with CO_2_ before being combined with the incubation buffer medium. In vitro, gas production was measured using the pressure transducer technique [35]. By mixing buffer solution (NH_4_HCO_3_ and NaHCO_3_), macro mineral, micro-mineral, and resazurin, the incubation medium was prepared [36] and then fluxed with CO_2_. To this buffer solution, adding a reducing agent and gassing with CO_2_ continued. When the pink colour disappeared, rumen liquor was added, and fluxing with CO_2_ continued. A sample (200 mg) of individual accession was weighed in 100 mL serum bottles in triplicate. For blank control, 3 bottles of serum were used with no substrate. Each sample and control received 50 mL of inoculum medium (buffer and rumen liquor) and was briefly gassed with CO_2_. The bottles were then sealed, and a gas pressure transducer was used to adjust the head-space gas pressure to zero on the LED display. The bottles were incubated at 39 °C for 24 h followed by measurement of the gas production (mL) with two times of repeat of entire incubation process.

#### 2.4.2 Measurement of methane

Methane produced at 24 h of fermentation was measured using gas chromatography flame ionization detector (GC-FID) (Nucon 5765 Microprocessor controlled GC, Okhla, New Delhi, India). The GC packed column of a stainless-steel with 2 m length and diameter of 2 mm. One mL of gas was manually injected at an inlet temperature of 100 °C using Hamilton syringe. The gas sample was then analysed in a GC-FID with column operating isothermally at 320 °C. The corrected methane values were corelated to the total gas to estimate its concentration [37]. The short-chain fatty acids (SCFA) estimated based on 24 h gas production using the previous method [38]. Furthermore, microbial mass (MBM) and partitioning factor (PF) were determined using the equation provided by Blümmel and his co-workers [39].

### 2.5 Ensiling of sugar-rich accessions

The 11 accessions namely, IG-02-695-1, IG-2045-1, IG-02-703, IG-99-198, IG-01-321-1, BS-1, IG02-713, IG99-181, IG02-695, IG-2041-2 and IG99-191 from the 19 selected sugar enriched accessions, which had adequate biomass were harvested from three different plots in September 2018 and wilted for 2 h to ensile. The harvested samples were manually chaffed into pieces of length size of 1–1.5 cm and placed inside of the plastic containers (25.5 cm × 13 cm, 5 kg volume), followed by manually pressed using hands and broad-base wooden rods to expel air. The containers were sealed with caps and adhesive tape and stored for ensiling. After 50 days, they were opened to assess the ensiling parameters.

### 2.6 Analysis for silage quality

Approximately 100 g sample of fresh silage was dried at 60 °C until it reached to the constant weight. The dry matter (DM) content calculated by the formula: estimated true DM (%) = 4.686 + (0.89 × oven DM %) [40]. After the dry matter, pH and lactic acid levels were measured, 20 g of fresh sample was mixed with 100 mL warm water in a beaker. This mixture was placed in a water bath shaker at 30 °C for 30 min, then filtered through filter paper. The pH of a portion of the filtrate was measured with a digital pH meter (Systronic 360), while the remaining filtrate was used to estimate lactic acid [41]. For lactic acid determination, 1 mL of silage extract was mixed with 0.05 mL of 4 % CuSO_4_ and 6 mL of concentrated H_2_SO_4_, added slowly drop by drop with the continuous shaking. The tubes were boiled in a water bath for 5 min, then cooled to room temperature. Next, 0.1 mL of *p*-hydroxyphenyl reagent was added drop by drop. The mixture was incubated in a shaker water bath at 30 °C for 30 min, and the resulting blue color was measured at 560 nm using UV-spectrophotometer (a LABINDIA3000).

### 2.7 Statistical analysis

Univariate statistics, such as means and ranges, were utilized to evaluate the variability among 92 Sehima nervosum (SN) accessions for various traits. Descriptive statistics were performed to focus on nutritional content, sugar, and biomass yield, aiming to identify sugar-rich accessions for further investigation. Broad-sense heritability (H2) for each forage quality trait was calculated following the method by Hill et al. [42]. After the initial screening, 19 accessions were selected for a comprehensive analysis of their chemical composition, sugar content, carbohydrate and protein fractions, energy values, digestibility, and in vitro gas and methane production. In the final phase, silage quality (pH, lactic acid, and DM content) was assessed based on the availability of sufficient biomass. To determine significance levels, one-way ANOVA was performed using SPSS 20.0, and Duncan's multiple-range test was used to compare trait means at *p* < 0.05 [43].

Additionally, a Hierarchical cluster analysis was performed on the standardized data to group the *Sehima* lines (92 accessions) using R statistical software. This method calculates a matrix of Euclidean distances among group means, generates a dendrogram showing the successive fusion of individuals, and concludes when all individuals within the same group form a cluster.

## 3 Results

### 3.1 Forage yield and nutritional variability in Sehima nervosum germplasm

The variance and range values indicate that the SN germplasm showed significant variability in chemical composition (including, proximates), sugar contents, other nutritional traits, and green fodder yield (GFY) (Table 1). Supplementary Table S1 provides the specific values for these traits for each accession. Notably, the GFY of the germplasm was higher post-rainy season, reaching 27.95 t ha^−1^, compared to 9.09 t ha^−1^ in the summer. The germplasm's CP content ranged from 3.14 % to 6.01 % DM, NDF content varied between 68.77 % and 80.14 % DM, ADF content ranged from 43.66 % to 54.31 % DM, cellulose content was between 24.97 % and 37.75 % DM, and lignin content varied from 3.95 % to 7.94 % DM. The average sugar content of *SN* germplasm was 63.34 mg g^−1^ and the values varied from 27.04 to 123.3 mg g^−1^. The mean values for DMI, DDM, and RFV were 1.63 %, 54.74 %, and 64.02 %, respectively. Similarly, the mean values for the germplasm's total digestible nutrients (TDN) were 41.19 %, digestible energy (DE) was 1.85 Mcal kg^−1^ DM, metabolizable energy (ME) was 1.52 Mcal kg^−1^ DM, net energy for maintenance (NE_M_) was 1.17 Mcal kg^−1^ DM, net energy for lactation (NE_L_) was 0.889 Mcal kg^−1^ DM, and net energy for gain (NE_G_) was 0.185 Mcal kg^−1^ DM.

**Table 1:** Descriptive statistics of 92 *Sehima nervosum* germplasms for forage quality and green fodder yield.

Parameters	Mean	SE	Minimum	Maximum	CV%
CP	4.28	0.07	3.14	6.01	14.68
NDF	73.82	0.25	68.77	80.14	3.26
ADF	48.98	0.23	43.66	54.31	4.6
cellulose	31.57	0.24	24.97	37.75	7.3
lignin	5.63	0.08	3.95	7.94	13.9
sugar mg g^−1^	63.34	2.02	27.04	123.32	30.51
GFY post rainy	27.95	1.52	2.2	58.08	52.18
GFY summer	9.09	0.62	0.48	30.8	65.01
Total GFY	37.04	1.91	3.12	83.95	49.5
DMI	1.63	0.01	1.50	1.75	3.26
DDM	50.74	0.19	46.60	54.89	3.49
RFV	64.02	0.36	56.14	72.00	5.38
TDN	41.19	0.31	34.26	48.13	7.19
DE	1.85	0.01	1.54	2.16	7.9
ME	1.52	0.01	1.26	1.77	7.19
NEM	1.17	0.01	0.96	1.37	7.37
NEL	0.89	0.01	0.72	1.06	8.16
NEG	0.18	0.01	−0.02	0.39	46.52

CP, Crude protein%; NDF, Neutral detergent fiber %; ADF, Acid detergent fiber %; GFY, Green fodder yield t/h.

Broad sense Heritability of forage quality traits in SN accessions ranged from 0.41 (lignin) to 0.98 (sugar), the given in Supplementary Table S2. Most of the forage quality traits had a broad-sense heritability (H^2^) more than 0.5, suggesting that these traits could be improved through phenotypic selection in the breeding programs. Even though the lignin had the lowest H^2^ (0.41) with the genetic variation statistically significant (p < 0.01). Hence, still there is potential to improve lignin content by selective breeding programs, however, the progress for the trait is anticipated to be less efficient and slower compared to other traits with higher heritability (H^2^) values.

**Fig. 2:**
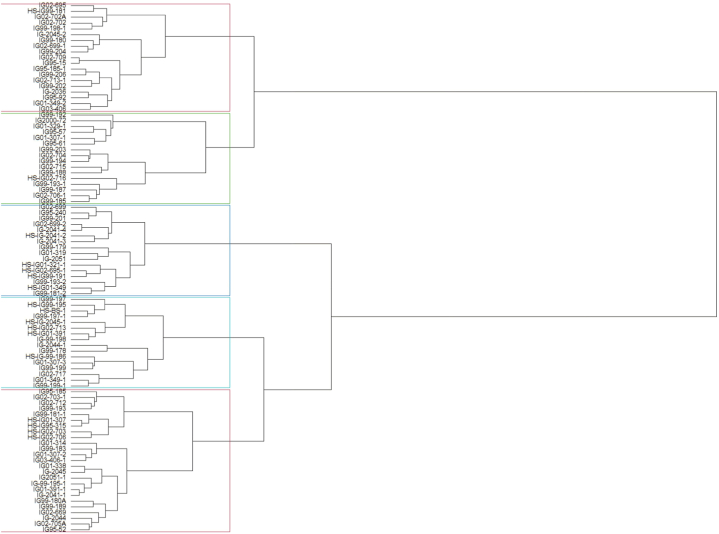
Dendrogram resulting from the cluster analysis performed on various biomass and forage quality traits of 92 *Sehima nervosum* accessions.

The dendrogram generated from the cluster analysis using the Euclidean method grouped the tested SN accessions into two main clusters based on dissimilarity matrices (Fig. 2). Main cluster “I” further divided into two sub-cluster comprised of 11 and 35 genotypes respectively. Similarly, main cluster “II” accommodated 48 genotypes further divided into three sub-clusters comprised of 14, 14 and 18 genotypes respectively. The selected all 11 high sugar genotypes are distributed in all three sub-cluster of cluster II. The tested genotypes exhibited a high degree of diversity, resulting in their classification into distinct clusters and supported by the range value of different traits indicated their suitability to choose the diverse lines from different clusters for further research in developing linkage maps and molecular markers, especially for forage quality traits.

### 3.2 Protein, fiber and sugar contents and S. nervosum accessions

The nineteen SN accessions, including IG-02-703, IG-01-349, IG-01-321-1, IG-99-195, IG-02-716, IG-02-695, IG-02-695-1, IG-02-669, IG-99-198, IG-95-315, IG-99-186, IG-01-319, IG-2041-2, IG-2045-1, IG-99-193, IG-01-307, IG-01-391, IG-02-713, and IG-99-191, all had sugar contents exceeding 70 mg g^−1^ DM, which is crucial for lactic acid production (Table 2). These values significantly differed (*p<0.05*) among the accessions. At 1st cut (during post-rainy season) accessions, mean sugar contents were higher (89.67) than the 2nd cut (during spring season) and were close to or above 70 mg g^−1^ DM except IG99-195 and IG99-191 (52.82 and 51.54 mg 100 g^−1^ DM). The accessions exhibited significant variation (*p<0.05*) in their CP, NDF, ADF, cellulose, and lignin content. The ranges were 4.71–9.01 % DM for CP, 67.66–77.62 % DM for NDF, 46.58–55.22 % DM for ADF, 33.12–38.01 % DM for cellulose, and 4.04–7.41 % DM for lignin. (Table 2). EE and OM also differed (*p<0.05*) between 1.12 and 4.04 % and 84.20–89.48 DM, respectively.

**Table 2:** Proximate compositions (%and sugar (mg g^−1^) contents of *S. nervosum* accessions.

Accessions	OM	CP	EE	NDF	ADF	cellulose	Lignin	Sugar	
								1st cut	2nd cut
IG-02-703	85.70^bcd^	9.63^h^	3.19^cde^	67.66^a^	53.44^gh^	36.91^defg^	7.41^g^	123.54^p^	100.71^j^
IG-01-349	86.12^bcd^	7.38^e^	3.38 ^def^	71.56^b^	48.00^b^	35.29^bcd^	4.75^b^	93.89^k^	87.31^g^
IG-01-321-1	87.74^ef^	8.67^g^	3.16^bcde^	74.36^efg^	51.38^f^	33.12^a^	6.23^ef^	105.7^n^	97.37^ij^
IG-99-195	86.76^cde^	7.92^f^	2.84^bc^	75.20^fgh^	53.26^gh^	37.21^efg^	6.16^ef^	83.18^ef^	52.82^a^
IG-02-716	87.14 ^de^	6.86^cd^	2.94^bcd^	76.11^h^	51.23^ef^	33.89^ab^	5.07^bc^	95.89^l^	95.02^h^
IG-02-695	86.35^bcde^	7.08^cde^	2.74^b^	75.46^gh^	49.53^cd^	35.40^bcd^	4.04^a^	73.37^b^	69.94^bcd^
IG-02-695-1	84.20^a^	6.78^c^	4.04^g^	71.82^bc^	50.22^de^	36.23^cdef^	5.18^bcd^	102.39^m^	81.76^f^
IG-02-669	86.87^cde^	9.01^g^	3.56^ef^	72.83^bcd^	53.86^gh^	37.67^fg^	6.38^f^	75.79^c^	74.78^de^
IG-99-198	88.64^fg^	7.34^de^	3.43^ef^	73.63^de^	55.22^i^	38.01^h^	7.40^g^	88.91^j^	80.12^ef^
IG-95-315	85.53^abc^	7.44^e^	3.71^fg^	73.68^de^	54.36^hi^	37.56^fg^	6.45^f^	82.66^ef^	81.81^f^
IG-99-186	85.74^bcd^	7.32^de^	3.61^efg^	71.70^bc^	46.58^a^	33.47^a^	5.17^bcd^	82.02^e^	74.98^de^
IG-01-319	89.48^g^	8.87^g^	1.26^a^	73.71^de^	48.34^bc^	35.91^bcd^	7.41^g^	72.00^a^	71.74^cd^
IG-2041-2	87.82^ef^	7.00^cde^	1.53^a^	73.03^cde^	53.47^gh^	35.58^cd^	6.45^f^	84.60^g^	70.56^bcd^
IG-2045-1	86.11^bcd^	7.06^cde^	1.12^a^	75.85^h^	52.01^g^	37.99^h^	5.87^def^	115.90^o^	93.43^h^
IG-99-193	86.60^bcde^	5.61^b^	1.26^a^	73.98^def^	53.16^gh^	35.82^cde^	6.42^f^	77.09^d^	72.81^cd^
IG-01-307	86.89^cde^	4.78^a^	1.36^a^	73.09^cde^	53.10^g^	35.64^cde^	6.10^ef^	85.91^h^	66.01^b^
IG-01-391	85.09^ab^	4.71^a^	1.36^a^	73.48 ^de^	49.30 ^cd^	34.63^abc^	5.39^bcd^	87.30^i^	67.56^bc^
IG-02-713	86.33^bcde^	7.03^cde^	1.28^a^	73.01^cde^	51.30^ef^	35.43^bcd^	5.63^cde^	89.98^j^	73.73^d^
IG-99-191	85.80^bcd^	5.14^a^	1.31^a^	77.62^i^	51.61^f^	35.57^cd^	6.36^f^	83.67^fg^	51.54^a^
Mean	86.58	7.14	2.48	73.58	51.55	35.86	5.99	89.67	77.05
SE	0.174	0.181	0.141	0.290	0.317	0.211	0.127	2.223	2.196
*p* value	<0.0001	<0.0001	<0.0001	<0.0001	<0.0001	<0.0001	<0.0001	<0.0001	<0.0001

OM, Organic matter %; CP, Crude protein%; EE, Ether extract %; NDF, Neutral detergent fiber %; ADF, Acid detergent fiber %. Superscripts a–p within columns differs significantly between rows at (*P* < 0.05).

### 3.3 Carbohydrate & protein fractions of SN accessions

In *SN* sugar-rich accessions, tCHO, structural carbohydrates (SC), and nonstructural carbohydrates (NSC) varied (*p<0.05*) from 72.81 to 80.75, 63.45 to 75.41 and 3.87–9.36 % DM, respectively (Table 3). The accessions exhibited significantly higher (*p<0.05*) carbohydrate fraction C_B2_ (67.89) compared to C_A_ (9.36), C_B1_ (4.18), and C_c_ (18.70 % tCHO), respectively. The protein fractions of *SN* accessions varied significantly (*p<0.05*), with mean values were 27.79 % DM for P_A_, 18.56 % DM for P_B1_, 16.99 % DM for P_B2_, 19.51 % DM for P_B3_, and 17.15 % DM for P_C_ (Table 3).

**Table 3:** The fractions of carbohydrate and protein of *S. nervosum* accessions.

Accessions	tCHO	SC	NSC	C_A_	C_B1_	C_B2_	C_C_	P_A_	P_B1_	P_B2_	P_B3_	P_C_
IG-02-703	72.81^a^	63.45^a^	9.36^e^	15.50^i^	6.45^g^	53.62^a^	24.42^i^	25.31^abc^	18.79^def^	12.27^ab^	29.04^g^	14.60^bcd^
IG-01-349	75.47^cde^	68.72^b^	6.75^bc^	11.76^fgh^	2.08^ab^	71.05^f^	15.10^b^	28.63^cdef^	21.60^fg^	11.27^a^	18.60^bcd^	19.9^ef^
IG-01-321-1	75.83^de^	70.34^cde^	5.49^ab^	10.13^ef^	3.40^bcd^	66.74^cde^	19.73^ef^	25.37^abc^	11.80^ab^	16.43^bcd^	30.34^g^	16.06^bcde^
IG-99-195	76.03^def^	72.16^ghi^	3.87^a^	8.18^cde^	3.06^abcd^	69.30^ef^	19.46^def^	25.52^abc^	20.36^f^	15.75^abc^	20.86^cde^	17.5^cdef^
IG-02-716	77.31^fgh^	73.45^ij^	3.86^a^	3.79^a^	5.82^fg^	74.65^g^	15.74^b^	24.10^ab^	12.66^abc^	24.52^f^	18.36^abcd^	20.36^f^
IG-02-695	76.52^efg^	72.51^fghi^	4.01^a^	6.14^abcd^	2.98^abcd^	78.20^h^	12.67^a^	21.83^a^	16.24^cde^	18.24^cde^	23.87^ef^	19.81^ef^
IG-02-695-1	73.36^ab^	68.99^bc^	4.36^a^	6.07^abc^	6.02^g^	70.97^f^	16.94^cd^	27.94^bcdef^	13.11^abc^	17.06^cde^	21.87^de^	20.02^ef^
IG-02-669	74.30^bc^	70.18^defg^	4.12^a^	7.39^bcde^	5.28^efg^	66.72^cde^	20.61^fg^	24.63^abc^	27.60^h^	18.33^cde^	15.15^ab^	14.29^bc^
IG-99-198	77.85^ghi^	70.83^defg^	7.02^bcd^	11.60^fgh^	3.90^cde^	61.69^b^	22.81^hi^	27.07^bcde^	13.65^abc^	21.14^def^	17.94^abcd^	20.19^ef^
IG-95-315	74.38^bc^	70.58^def^	3.79^a^	4.31^a^	7.95^h^	66.93^cde^	20.81^fgh^	30.2^defg^	11.16^a^	16.93^bcde^	22.31^de^	19.35^ef^
IG-99-186	74.81^cd^	69.72^bcd^	5.09^a^	9.06^def^	3.34^bcd^	70.99^f^	16.60^cd^	31.28^fg^	26.41^h^	15.25^abc^	18.40^abcd^	8.67^a^
IG-01-319	79.34^jk^	70.98^defgh^	8.36^cde^	11.35^fg^	5.03^efg^	61.19^b^	22.42^ghi^	29.99^defg^	24.47^gh^	14.75^abc^	16.13^abc^	14.65^bcd^
IG-2041-2	79.29^ijk^	70.77^defg^	8.52^de^	13.66^ghi^	2.18^ab^	64.62^c^	19.53^def^	26.54^bcd^	24.26^gh^	17.03^cde^	13.50^a^	18.66^def^
IG-2045-1	77.93^hij^	73.83^j^	4.09^a^	6.02^abc^	4.36^def^	71.53^f^	18.09^cde^	31.44^fg^	22.31^fg^	17.57^cde^	13.76^ab^	14.93^bcd^
IG-99-193	79.73^kl^	72.03^fghi^	7.71^cd^	12.00^fgh^	2.58^abc^	66.09^cde^	19.33^def^	30.81^efg^	15.76^cd^	18.55^cde^	15.24^ab^	19.64^ef^
IG-01-307	80.75^l^	71.32^efgh^	9.43^e^	14.43^hi^	1.57^a^	65.87^cd^	18.13^cde^	26.49^bcd^	15.20^bcd^	21.23^ef^	16.10^abc^	20.98^f^
IG-01-391	79.02^ijk^	72.03^fghi^	6.99^bcd^	9.04^def^	4.15^de^	70.43^f^	16.38^cd^	28.6^cdefg^	24.74^gh^	15.50^abc^	13.75^ab^	17.40^cdef^
IG-02-713	78.02^hij^	70.81^defg^	7.21^cd^	10.06^ef^	4.06^cde^	68.56^def^	17.33^bcd^	32.10^g^	19.70^ef^	16.96^bcde^	18.54^bcd^	12.70^b^
IG-99-191	79.35^jk^	75.41^k^	3.94^a^	4.83^ab^	5.13^efg^	70.80^f^	19.24^def^	30.13^defg^	12.78^abc^	14.09^abc^	26.91^fg^	16.08^bcde^
Mean	76.95	70.95	6.00	9.23	4.18	67.89	18.70	27.79	18.56	16.99	19.51	17.15
SE	0.317	0.330	0.282	0.485	0.235	0.726	0.398	0.443	0.738	0.485	0.719	0.484
*P* value	<0.0001	<0.0001	<0.0001	<0.0001	<0.0001	<0.0001	<0.0001	<0.0001	<0.0001	<0.0001	<0.0001	<0.0001

tCHO, Total carbohydrates % DM; NSC, Non-structural carbohydrates % DM; SC, Structural carbohydrates % DM; C_A_, Rapidly degradable sugars % tCHO; C_B1_, Intermediately degradable starch and pectins % tCHO; CB2, Slowly degradable cell wall % tCHO; CC, Unavailable/lignin-bound cell wall % tCHO; P_A_, Non-protein nitrogen; P_B1_, Buffer-soluble protein; P_B2_, Neutral detergent-soluble protein; P_B3_, Acid detergent-soluble protein; P_C_, Indigestible protein; Superscripts a–k within columns differ significantly between rows at (*p* < 0.05).

### 3.4 Energy value of SN accessions

In sugar-rich *SN* accessions, there was significant variation (*p<0.05*) in TDN, DE, and ME, with ranges of 33.07–44.32 %, 1.46–1.87 kcal g^−1^ DM, and 1.20–1.54 kcal g^−1^ DM, respectively (Table 4). The energy efficiency for maintenance (NE_M_), lactation (NE_L_), and Growth (NE_G_) also varied significantly (*p<0.05*) among these accessions, with mean values of 0.969, 0.808, and 1.37 kcal g^−1^ DM, respectively.

**Table 4:** Energy contents of *S. nervosum* accessions.

Accessions	TDN%	DE Kcal g^−1^	ME Kcal g^−1^	NE_L_ Kcal g^−1^	NE_M_ Kcal g^−1^	NE_G_ Kcal g^−1^
IG-02-703	35.40^bc^	1.56^ab^	1.28^bc^	0.747^bc^	0.897^bc^	0.016^bc^
IG-01-349	42.47^h^	1.87^h^	1.54^h^	0.921^h^	1.103^h^	0.222^h^
IG-01-321-1	38.07^d^	1.68^d^	1.38^d^	0.813^d^	0.975^d^	0.094^d^
IG-99-195	35.63^bc^	1.57^ab^	1.29^bc^	0.753^bc^	0.904^bc^	0.023^bc^
IG-02-716	38.26^de^	1.69^de^	1.39^de^	0.817^de^	0.981^de^	0.100^de^
IG-02-695	40.48^fg^	1.79^fg^	1.47^fg^	0.877^fg^	1.045^fg^	0.164^fg^
IG-02-695-1	39.59^ef^	1.75^ef^	1.43^ef^	0.850^ef^	1.019^ef^	0.138^ef^
IG-02-669	34.84^bc^	1.54^ab^	1.26^bc^	0.734^bc^	0.881^bc^	0.000^bc^
IG-99-198	33.07^a^	1.46^a^	1.20^d^	0.690^a^	0.830^a^	0.051^a^
IG-95-315	34.19^ab^	1.51^ab^	1.24^ab^	0.718^ab^	0.863^ab^	0.018^ab^
IG-99-186	44.32^i^	1.95^i^	1.60^i^	0.966^i^	1.156^i^	0.275^i^
IG-01-319	42.03^gh^	1.85^gh^	1.52^gh^	0.910^gh^	1.090^gh^	0.209^gh^
IG-2041-2	35.35^bc^	1.56^ab^	1.28^bc^	0.746^bc^	0.896^bc^	0.015^bc^
IG-2045-1	37.26^d^	1.64^c^	1.35^d^	0.793^d^	0.951^d^	0.070^d^
IG-99-193	35.75^bc^	1.58^c^	1.29^bc^	0.756^bc^	0.908^bc^	0.027^bc^
IG-01-307	35.84^c^	1.58^c^	1.30^c^	0.758^c^	0.910^c^	0.029^c^
IG-01-391	40.78^fg^	1.80^fg^	1.48^fg^	0.879^fg^	1.054^fg^	0.173^fg^
IG-02-713	38.18^de^	1.68^d^	1.38^de^	0.815^de^	0.978^de^	0.097^de^
IG-99-191	37.77^d^	1.67^d^	1.37^d^	0.805^d^	0.966^d^	0.085^d^
Mean	37.86	1.67	1.37	0.808	0.969	0.088
SE	0.412	0.018	0.015	0.010	0.012	0.012
*P* value	<0.0001	<0.0001	<0.0001	<0.0001	<0.0001	<0.0001

TDN, Total digestible nutrients %; DE, Digestible energy Mcal kg^−1^; ME, Metabolizable energy Mcal kg^−1^; NE_L_, Net energy for lactation Mcal kg^−1^; NE_G_, Net energy for growth/gain Mcal Kg^−1^; NE_M_, Net energy for maintenance Mcal kg^−1^. Superscripts a–i within columns differ significantly between rows at (*p* < 0.05).

### 3.5 Intake, feed value, and digestible nutrients of SN accessions

In sugar-rich SN accessions, the values for DMI, DDM, and RFV varied significantly (*p<0.05*), ranging from 1.55 to 1.77 %, 45.88–52.61 %, and 57.96–68.24 %, respectively (Table 5). The digestible nutrients varied significantly (*p<0.05*), DCP, dEE, and dCHO had mean values of 3.60, 2.40, and 45.25 %, respectively. Accession IG-01-391 had the lowest contents of DCP, dEE, and dCHO, with values of 1.40, 1.30, and 39.09 %, respectively.

**Table 5:** Intake, feed value and nutrients digestibility of *S. nervosum* accessions.

Accessions	DMI%	DDM%	RFV%	DCP	dEE	dCHO
IG-02-703	1.77^h^	47.27^bc^	64.98^h^	0.060^f^	0.032^cd^	439.11^bcde^
IG-01-349	1.68^g^	51.51^h^	66.94^i^	0.039^cd^	0.033^cd^	476.95^ef^
IG-01-321-1	1.61^cdef^	48.87^d^	61.14^def^	0.051^e^	0.031^cd^	472.05^def^
IG-99-195	1.60^bcd^	47.41^bc^	58.64^abc^	0.044^de^	0.028^c^	453.55^cde^
IG-02-716	1.58^b^	48.99^de^	59.86^bcd^	0.034^b^	0.029^c^	470.45^def^
IG-02-695	1.59^bcd^	50.32^fg^	61.92^efg^	0.036^bcd^	0.027^cd^	529.60^g^
IG-02-695-1	1.67^g^	49.78^ef^	64.46^h^	0.033^bc^	0.040^e^	508.19^fg^
IG-02-669	1.65^fg^	46.94^bc^	59.94^bcd^	0.054^e^	0.035^d^	481.61^ef^
IG-99-198	1.63^ef^	45.88^a^	57.96^a^	0.038^bcd^	0.034^d^	416.90^abc^
IG-95-315	1.63^ef^	46.55^ab^	58.77^abc^	0.039^cd^	0.037^e^	421.12^abc^
IG-99-186	1.67^g^	52.61^i^	68.24^i^	0.038^cd^	0.036^d^	472.47^def^
IG-01-319	1.63^ef^	51.24^gh^	64.66^g^	0.053^e^	0.012^a^	461.22^cde^
IG-2041-2	1.64^g^	47.25^bc^	60.17^bcd^	0.035^bcd^	0.015^b^	438.35^bcde^
IG-2045-1	1.58^bc^	48.39^d^	59.32^abcd^	0.036^bcd^	0.011^a^	448.18^bcde^
IG-99-193	1.62^def^	47.48^bc^	59.69^abcd^	0.022^a^	0.012^a^	451.76^bcde^
IG-01-307	1.64^fg^	47.54^c^	60.49^cde^	0.014^a^	0.013^a^	431.38^abcd^
IG-01-391	1.63^ef^	50.49^fg^	63.91^gh^	0.014^a^	0.013^a^	390.94^a^
IG-02-713	1.64^fg^	48.94^de^	62.34^fg^	0.035^bc^	0.012^a^	408.14^ab^
IG-99-191	1.55^a^	48.70^d^	58.35^ab^	0.018^a^	0.013^a^	420.87^abc^
Mean	1.63	48.75	61.67	0.036	0.024	452.25
SE	0.007	0.247	0.410	0.002	0.0015	5.127
*P* value	<0.0001	<0.0001	<0.0001	<0.0001	<0.0001	<0.0001

DMI, Dry matter intake %; DDM, Digestible dry mater% DM; RFV, Relative feed value%; DCP, Digestible crude protein kg kg^−1^ DM; dEE, digestible ether extract; dCHO, Digestible carbohydrates. Superscripts letters (a–i) within columns differ significantly at (*p* < 0.05).

### 3.6 Gas production, fermentation characteristics, and CH_4_ production from SN accessions

The in vitro gas fermentation parameters showed significant variations (*p<0.05*). The ranges were 66.45–107.76 for degraded dry matter (mg 200 mg^−1^), 3.43–5.18 for partition factor-PF (mg mL^−1^), 1.43–3.21 for short chain fatty acids (SCFA), 131.10–299.12 for microbial mass (MBM) and 0.36–0.68 for efficiency for microbial mass production (EMBP). The accession IG-01-391 had higher PF, MBM, and EMBP values and but lower SCFA. Total gas and CH_4_ production also varied (*p<0.05*) among SN accessions, with mean values of 101.56 and 10.95-mL g^−1^ DM, respectively (Table 6). CH_4_ production (mL g^−1^ DDM) ranged from 20.51 to 32.65 across accessions. Among the sugar-rich accessions, IG-99-195 had the lowest CH_4_ % TG (7.91), while IG-01-391 had the highest (14.97).

**Table 6:** In vitro total gas and methane production of *Sehima* accessions.

Accessions	OMD%	ME	PF	SCFA	MBM	EMBP	CH_4_ mL g^−1^	CH_4_ mL g^−1^ DDM	Total gas mL g^−1^	CH_4_%TG
IG-02-703	43.97^bcde^	5.14^bcd^	4.55^bc^	1.77^abc^	178.80^abcde^	0.50^cde^	8.02^a^	22.56^abc^	79.84^abc^	10.04^bcde^
IG-01-349	47.76^ef^	5.80^efg^	4.10^ab^	2.44^efg^	205.91^cdef^	0.46^abcd^	13.07^e^	29.14^def^	109.89^efg^	11.89^ef^
IG-01-321-1	47.26^def^	5.85 fg	3.66^ab^	2.44^efg^	160.40^abc^	0.40^abc^	10.56^bcd^	26.48^abcdef^	110.24^efg^	9.67^abcd^
IG-99-195	45.41^cde^	5.49^cdefg^	3.51^a^	2.22^def^	131.10^a^	0.37^ab^	7.93^a^	22.58^abc^	100.02^def^	7.91^a^
IG-02-716	47.11^def^	5.79^efg^	3.74^ab^	2.56^fg^	173.22^abcd^	0.40^abc^	11.94^cde^	28.02^bcdef^	115.30^fg^	10.41^bcde^
IG-02-695	53.03^f^	6.61^h^	3.65^ab^	3.21^h^	210.51^cdef^	0.40^abc^	16.34^f^	31.12^ef^	144.93^h^	11.29^cdef^
IG-02-695-1	50.90^g^	6.10^gh^	3.43^a^	2.71^g^	148.27^ab^	0.36^a^	12.27^de^	29.38^def^	122.44^g^	10.06^bcde^
IG-02-669	48.22^ef^	5.92^fg^	3.68^ab^	2.48^efg^	164.42^abc^	0.40^abc^	13.04^e^	31.84^ef^	111.89	11.68^cdef^
IG-99-198	41.74^abc^	5.12^bcd^	3.86^ab^	2.08^efg^	147.27^ab^	0.42^abc^	10.34^abcd^	29.17^def^	93.99	11.24^cdef^
IG-95-315	42.17^abc^	4.89^abc^	5.17^c^	1.85^abcd^	228.59^ef^	0.55^de^	9.66^abc^	23.45^abcd^	83.65^abcd^	11.43^cdef^
IG-99-186	47.31^def^	5.69^defg^	4.20^ab^	2.50^efg^	221.33^def^	0.47^bcd^	12.12^cde^	25.95^abcde^	112.75^efg^	10.75^cde^
IG-01-319	46.17^bcde^	5.84^fg^	4.25^ab^	2.58^fg^	238.17^g^	0.48^bcde^	10.16^abcd^	20.51^a^	116.31^fg^	8.67^ab^
IG-2041-2	43.89^bcde^	5.35^bcdef^	3.9^ab^	2.33^efg^	167.82^abc^	0.42^abc^	9.89^abcd^	24.74^abcd^	105.17^efg^	9.55^abc^
IG-2045-1	44.88^bcde^	5.33^bcdef^	4.06^ab^	2.17^cdef^	182.02^abcde^	0.45^abcd^	11.65^cde^	29.54^def^	97.92^cdef^	11.88^ef^
IG-99-193	45.24^bcde^	5.45^cdefg^	3.76^ab^	2.36^efg^	162.61^abc^	0.41^abc^	10.70^bcde^	27.00^bcdef^	106.60^efg^	10.09^bcde^
IG-01-307	43.19^abcd^	5.17^bcde^	4.15^ab^	2.14^cdef^	188.96^bcdef^	0.47^bcd^	11.35^cde^	28.42^cdef^	96.65^cdef^	11.76^cdef^
IG-01-391	39.15^a^	4.38^a^	6.99^d^	1.43^a^	299.12^h^	0.68^f^	9.66^abc^	21.95^ab^	64.70^a^	14.97^g^
IG-02-713	40.87^ab^	4.74^ab^	5.18^c^	1.65^ab^	220.99^def^	0.57^e^	8.79^ab^	22.78^abc^	74.67^ab^	11.78^def^
IG-99-191	42.15^abc^	4.90^abc^	3.96^ab^	1.83^abcd^	140.98^ab^	0.44^abc^	10.54^bcd^	32.65^g^	82.62^abcd^	12.87^f^
Mean	45.29	5.45	4.20	2.25	187.92	0.46	10.95	26.70	101.56	10.94
SE	0.518	0.078	0.011	0.055	5.617	0.011	0.268	0.552	2.463	0.218
*P* value	<0.0001	<0.0001	<0.0001	<0.0001	<0.0001	<0.0001	<0.0001	<0.0001	<0.0001	<0.0001

OMD, Organic matter digestibility%; ME, Metabolizable energy Mj Kg^−1^ DM; PF, Partition factor mg of in vitro degraded dry matter to ml of gas produced; SCFA, Short-chain fatty acid mmole g^−1^ DM; MBM, Microbial mass mg g^−1^ DM; EMBP, Efficiency of microbial protein production mg mg^−1^; CH_4_, Methane; DDM, Degraded dry matter; TG, Total gas; Superscripts letters (a–h) within columns differ significantly between rows at (*p* < 0.05).

### 3.7 Silage composition

The DM contents of silage prepared from 11 SN accessions differ (*p<0.05*) and were highest for IG02-695 (36.02) and lowest for BS-1 (22.95 %), respectively (Table 7). Also, the pH value and lactic acid levels of evaluated SN accessions differed significantly (*p<0.05*) and ranged from 4.57 to 5.61 and 0.094–1.530 % DM, respectively.

**Table 7:** Dry matter, pH and lactic acid contents of *Sehima* accessions silage.

Accessions	DM	pH	LA
IG-02-695-1	28.30^c^	5.28^d^	1.530^c^
IG-2045-1	32.07^fg^	4.85^b^	0.094^a^
IG-02-703	30.60^e^	4.57^a^	0.490^b^
IG-99-198	26.73^b^	5.43^e^	1.774^c^
IG-01-321-1	32.81^g^	5.28^d^	0.250^ab^
BS-1	22.95^a^	5.06^c^	0.456^b^
IG02-713	31.61^f^	4.58^a^	0.165^a^
IG99-186	29.35^d^	5.61^f^	0.146^a^
IG02-695	36.02^h^	5.24^d^	0.109^a^
IG-2041-2	32.22^fg^	5.25^d^	0.150^a^
IG99-191	31.47^f^	4.77^b^	0.213^ab^
Mean	30.38	5.08	0.49
SE	0.589	0.057	0.102
*P* value	<0.0001	<0.0001	<0.0001

LA, Lactic acid %DM; DM, Dry matter. Superscripts a–h within columns differs significantly between rows at (*P* < 0.05).

## 4 Discussion

### 4.1 Chemical composition and yield analysis

The primary indicator used to assess the nutritive value of any feed or fodder is the chemical composition. The mean CP content of the *SN* germplasm was lower at 4.28 % DM, whereas the identified sugar enriched accessions showed more CP content than 7.0 % DM, which is necessary for microbial activity in the rumen [44]. It is known that crude protein is considered the crucial measure for nutritional quality for any accession or germplasm [45]. The exceeded the 8.0 % DM level in the sugar enrich accessions were considered optimum for growing beef cattle maintenance [46]. The NDF, ADF, EE, lignin, and ash contents of *SN* 7.0, 73.7, 44.9, 6.5, and 8.8 % [47] were aligned in the scale of studied accessions values, whereas CP content was contradict with lower value (4.28 % DM).

Ludwig and his co-workers reported CP (6.1 %) and NDF (74.6 %) of SN from open grassland, and these values lie within values of both germplasm and sugar-rich accessions [48]. Similarly, Reddy and Reddy reported that crude protein, ether extract, neutral detergent fiber, acid detergent fiber, cellulose, hemicelluloses, and, lignin of *SN* chopped grass/ground pellets were 3.5, 2.0, 74.2, 53.4, 32.3, 20.8 and 17.2 %, respectively, which lies within the range of *SN* accessions evaluated in the current study [49]. In another study, lower CP (4.10 %) and similar NDF (70.95), ADF (44.78), cellulose (33.50), and lignin (96.20 % DM) were reported, aligning with values presented in this study [50]. Genetic manipulation through the sexual transfer of genetic information between SN genomes is challenging due to its facultative apomictic and polyploid nature [22]. Due to the predominantly apomictic nature of the species, the most common method for developing desired lines involves collecting naturally occurring variability over time and selecting among the collected germplasm for desirable traits. The green biomass yield is the most important trait for developing high-yielding cultivars [51]. Among the evaluated 92 germplasm accessions, the highest range of green fodder yield was obtained up to 58.08 t ha^−1^ in the rainy and up to 30.80 t ha^−1^ in the summer season. However, the improved cultivar produced up to 30–40 t ha^−1^ green biomass yields under rain-fed conditions [52]. This indicates that potential accessions for high biomass yield are present in evaluated germplasm set.

Information on carbohydrate and protein fractions of forage crops can increase nutrient utilization efficiency and help to decide the type of supplementation [53] to synchronize energy and protein for maximizing microbial production [54]. Sugar-rich *SN* accessions tCHO (72.81–80.75 % DM) lie within the values (73.0–79.5 % DM) of seven tropical grasses reported at 56 days of cutting [55]. The total carbohydrates (tCHO) and non-fiber carbohydrates (NFC) in grasses such as *Cynodon dactylon*, *Brachiaria brizantha*, and *Panicum maximum* ranged from 72.8 % to 82.7 % DM and 2.0 %–9.0 % DM, respectively. [56], corroborating our values. On the other hand, the NSC reported in fifteen tropical grasses was higher than our *SN* accessions NSC values [57]. The tCHO of four tropical grasses [58] and were close to our tCHO, while NSC contents were higher than sugar-rich accessions NSC values. Variability in carbohydrate C_A + B1_, C_B2,_ and C_C_ fractions of different grass species due to growing season and cutting age has been reported [56,59]. In the present study, *SN* accessions to higher carbohydrate C_B2_ and C_C_ fractions may be due to higher NDF and lignin contents because forages having high NDF exhibits higher C_B2_ fractions, and higher C_C_ can be partly ascribed to higher lignin in NDF [60].

Protein fractions of grasses vary with species and growth age [61]. The P_C_ fraction in sugar-rich *SN* accessions varied, with a mean value of 17.15 % CP, slightly higher than the previously reported range of 10.0–15.0 % CP [62]. The higher P_C_ fraction observed in this study may be attributed to the increased lignin content in SN accessions. This protein fraction includes nitrogen that is insoluble in acid detergent solution and is linked with lignin, tannin-protein complexes, and Maillard products, rendering it unavailable and indigestible in both the rumen and intestines. In our study, P_C_ fraction ranged significantly (*p<0.05*) from 8.67 to 20.98, similar to the reported values of 9.0–18.0 % CP [63,64]. Additionally, studied *Pc* fraction values fell within range of 5.4–27 %, reported for tropical grasses earlier [65]. Brandstetter and his co-workers evaluated Jiggs Bermuda grass across 4 seasons, reported higher PA (40.7–55.0) and lower P_B1_, P_B2_, P_B3_, and P_C_ values (13.8–13.9, 10.0–15.7, 12.5–14.8 and 8.0–11.5%CP) than our values of *SN* accessions [59]. *C. dactylon, B. brizantha,* and *P. maximum* grasses P_A_, P_B1+B2_, P_B3,_ and P_C_ fractions at three different cuts (28, 35 and 54 days) varied between 16.4 and 28.7, 25.1–53.8, 11.6–34.9 and 9.1–18.2 % CP, respectively [56] substantiates protein fraction values of the present study.

### 4.2 Sugar contents of sehima germplasm

The available free sugar within the forage plant is essential for animal nutrition and silage fermentation [66]. Sugar content plays a vital role in the ensiling process of fodder, serving as the primary nutrient source for microbes to start fermentation and produce lactic acid. Ideally, the sugar content should be between 50 and 86 mg g^−1^ DM to ensure effective fermentation. Adequate sugar levels not only support microbial activity but also enhance the preservation and nutritional quality of the silage [12]. The sugar content of SN germplasm ranged from 27.04 to 123.32 mg g^−1^ DM, showing significant variation among sugar-rich accessions. The average soluble sugar content of the germplasm was 63.34 mg g^−1^ DM, which is below the desired level for effective ensiling. In contrast, the mean sugar content of sugar-rich accessions at both cuts was higher, at 89.67 and 77.05 mg g^−1^ DM, surpassing the 70 mg g^−1^ DM required for optimal ensiling. Tropical grasses typically have low sugar and water-soluble carbohydrate (WSC) levels, which can limit lactic acid production during ensilage, leading to lower quality silage. Ensuring adequate sugar levels is crucial for producing high-quality silage with sufficient lactic acid content [67]. The water-soluble carbohydrate (WSC) content of six tropical grasses ranged from 12.6 to 98.8 mg g^−1^ DM, which aligns with the sugar content observed in the evaluated germplasm and selected accessions [68]. Piltz and Kaiser conducted studies on various tropical grasses and reported their WSC contents. Specifically, they found that Kikuyu grass had a WSC content of 45 mg g^−1^ DM, Seteria grass had 48 mg g^−1^ DM, Rhodes grass had 86 mg g^−1^ DM, Signal grass had 99.3 mg g^−1^ DM, Napier grass had 31 mg g^−1^ DM, Guinea grass had a certain value, and Paspalum grass had another value. These findings highlight the variability in WSC content among different grass species, which is crucial for understanding their potential for lactic acid production during ensiling [69]. *Pennisetum clandestinum, Paspalum dilatatum, Panicum dichotomiflorum, Digitaria sanguinalis,* and *Eleucine indica* subtropical grasses soluble sugar contents in leaves varied between 43 and 66, 64–86, 85–97, 57–63 and 38–80 mg g^−1^ DM in different areas/location of their growth [70]. These values lie within the sugar contents of the present study. Cunha et al. reported significant variability in the sugar content of 95 accessions of elephant grass with range highest to lowest sugar content was 6.96 to 4.03 % Brix, respectively [71].

### 4.3 Energy contents and other quality traits (intake, feed value, and digestible nutrients) of SN accessions

Energy is the single most critical nutrient of animal diet required for basal metabolism, growth, production, and reproduction. The total digestible nutrients (TDN), digestible energy (DE), and metabolizable energy (ME) of ten tropical grasses ranged from 34.2 % to 60.9 % DM, 5.92 to 11.26 Mj kg^−1^ DM, and 4.85 to 9.23 Mj kg^−1^ DM, respectively [72] substantiates *SN* germplasm (34.26–48.13%DM, 1.54–2.16 and 1.26–1.77 Mcal kg^−1^ DM) and accessions values (33.07–44.32%DM, 1.46–1.95 and 1.60 Mcal kg^−1^ DM). Higher TDN, DE, and ME values for IG99-196, IG-01-349, and IG-01-319 accessions might be due to their lower ADF contents (46.58, 48.00, and 48.34%DM) as higher ADF reduces nutrient utilization in forages [73]. The average metabolizable energy (ME) values for SN germplasm and accessions, which are 1.52 and 1.37 Mcal kg^−1^ DM respectively, were found to be lower compared to the ME values of 17 grass samples, which ranged from 7.7 to 13.6 MJ kg^−1^ DM [38]. and in sufficient to fulfil energy requirements to maintain growth of cattle (8.8 Mj kg^−1^ DM) [74]. Several factors, viz. species, season, growth stage, and environmental conditions, influence the ME contents of tropical grasses [75]. Net energy is the most precise measure for expressing the useable energy content of feeds and the energy requirements of animals. The efficiency of NE in feed/fodder is lower for growth (N_EG_) compared to maintenance (N_EM_) and lactation (N_EL_). Consequently, feeds/fodders have higher NE values for maintenance and lactation than for growth or fattening. The NEL values for Napier and Pangola grass (1.05 and 1.16 Mcal kg^−1^ DM, respectively) [75] are higher than mean values of both *SN* germplasm (0.89) and sugar-rich SN accessions (0.81 Mcal per kg DM). NE_L_ values for accessions IG99-124, IG96-358, IG96-96 and IG96-98 are nearly identical with earlier reports [75]. The mean NE_M_ values for both *SN* germplasm and, sugar-rich accessions were below than the recommended for an adult beef cow [76].

Forage quality traits primarily encompass nutrient contents, digestibility, anti-quality factors, and how much animals consume (intake). Many workers [[77], [78], [79]] have proposed indices using feed/fodder chemical constituents to measure forage quality. Intake of an important forage quality trait primarily measures the nutrients available to animals, which varies with diet composition and animal species. Variability in DMI of *SN* germplasm and sugar-rich accessions (1.50–1.75 % and 1.55–1.77 %) might be ascribed to differences in NDF contents. Feeds/forages NDF contents >60.0–65.0 % DMhave a negative impact on intake [25], and mean values of SN germplasm and sugar-rich accessions (73.82 and 73.58 % DM) were above this level. Tropical grasses *Brachiaria ruzizinensis, Panicum maximum,* and *Pennisetum purpureum* DMI at the flowering stage differed from 30.0 to 54.0 g kg^−1w075^ in sheep fed ad lib [80]. Sudan grass and Elephant grass DMI (2.33 and 2.25 %) in goats recorded by a study [81] was higher than our calculated dry matter values for *SN* germplasm and sugar-rich accessions. Digestibility is one of the key factors in measuring forage/feed nutritive value and relates to nutrient contents and energy. Feeding forages with 50 % digestibility or more can fulfil grazing ruminants’ energy needs [82], and our samples DDM values are close to this value. The variability in digestible dry matter (DDM) for SN germplasm (46.60–54.89 %) and sugar-rich accessions (45.88–52.61 %) can be attributed to differences in their acid detergent fiber (ADF) and lignin contents. This is because the nature and quantity of cell wall and cell contents influence the degradability of dry matter in forages [44]. Warm season forages in large less digestible (45–66 %) than cool season forages (49–81 %) [83].

Relative feed value (RFV) combines intake and forage digestibility into one unit. Significant differences in RFV of eleven cool-season and four warm-season grasses between 71.5-130 % and 88–165 % reported [84] were higher than our RFV values of *SN* germplasm and sugar-rich accessions (56.14–72.00 and 57.96–68.24 %). The relative feed value evaluated over the years (3–5 years) varied with individual grasses like *Brachiaria decumbens* (74.83–84.17)*,* Dallis (68.0–82.1), Bermuda (75.0–93.7), and Rhodes grasses (72.8–86.7 %) [85,86] were also higher than our RFV values. The present study's lower RFV values of SN germplasm and sugar-rich accessions possibly because of higher value of ADF and NDF. In a study RFV values of 15 grass species from lowland and upland were reported between 53.88 and 73.16 %, with a mean value of 60.9 % corroborating our RFV values of germplasm and accessions [87].

### 4.4 In vitro fermentation, gas and methane production

Gas production measures microbial activity and OM degradability and ME contents of a forage/diet. The OMD (39.15–53.03 %) and ME contents (4.38–6.61 MJ kg^−1^ DM) of *SN* sugar-rich accessions were lower than OMD (42.0–73.0 %) and ME values (5.8–10.2 MJ kg^−1^ DM) of grass species reported [88]. Gobena et al. reported higher ME contents (6.05–8.34 Mj kg^−1^ DM) of 15 grass species than our ME values [87]. The ME contents of evaluated *SN* accessions must be revised to fulfill growing beef cattle's maintenance energy requirements (8.8 MJ kg^−1^ DM) [74]. Several workers [89,90] have recorded OMD and ME contents of different grass species. The lower OMD and ME contents for *SN* accessions may be ascribed to their higher NDF, ADF, cellulose, and lower gas production.

In a study is suggested that PF values for roughages from 2.75 to 4.45 mg mL^−1^, that shows YATP range 10–40 [39]. The PF values (3.8–4.3) of tropical grasses [91] were consistent with our *SN* accessions PF values baring IG-95-315 (5.17), IG-01-391 (6.99) and IG-02-713 (5.18) accessions. The differences in MBM and EMBM of sugar-rich *SN* accessions may be attributed to variability in their gas production values. Higher PF tended to increase MBM as it measures microbial production efficiency. The higher MBM and EMBM for IG-01-391 (299.12 mg g^−1^and 0.68 mg g^−1^) may be attributed to its higher gas production. Higher SCFA for IG-02-716, IG-02-695, IG-02-695-1, and IG01-319 (2.56–3.21) may be ascribed to their higher gas production as high positive correlation between gas production and SCFA concentration for six tropical grasses reported [92]. The lowest SCFA for IG-02-391 and IG-02–713 may be partially attributed to their lower dCHO contents (390.94 and 408.14 g kg^−1^ DM). Further, higher OMD reflected in increased SCFA of these genotypes is consistent with the observations earlier study [93].

Gas production indicates carbohydrate and protein degradation from a feed/forage [35]. The i*n vitro* gas production from 4 tropical grasses, ranging from 122 to 170 mL g^−1^ DM after 24 h of incubation, was relatively higher than the mean value of 101.56 mL g^−1^ DM for SN sugar-rich accessions [94]. It was also comparable to the gas production values of accessions IG-02-695 (144.93 mL g^−1^ DM) and IG-02-695-1 (122.44 mL g^−1^ DM). The total gas and methane (CH_4_) production from twenty-four grass species ranged from 94 to 232 mL g^−1^ DM and 26–43 mL g^−1^ DM, respectively [95]; were also higher than our methane values, while all the accessions except IG-02-703, IG-99-98, IG-01-391, IG-02-713, and IG-99-191 had gas production >95 mL g^−1^ DM. Contrary CH_4_ production (4.02–11.70 mL g^−1^ DM) from fermentation of 16 grasses at 24 h reported in a study [96] was lower than our CH_4_ values (7.93–16.34 ml g^−1^ DM). Methane production and CH_4_% total gas from sixteen tropical grasses varied (*p<0.001*) from 4.38 to 12.7 mL g^−1^ and 4.60–9.18 % [97] partially substantiates our CH_4_ production and CH_4_% total gas results. The ratio of CH_4_ to total gas of grasses varied from 0.107 to 0.178 [95], and our values fall within this range except IG-01-321-1, IG-99-196, IG-01-319 and IG2041-2. The differences in gas and CH_4_ production amongst the sugar-rich *SN* accessions may be attributed to variability in their chemical composition and degradability [88,98].

### 4.5 Silage quality

High-quality silage is difficult from tropical forages is challenging due to low soluble sugars and insufficient lactic acid bacteria [16]. Key factor influencing silage quality includes the forage's stage of maturity, exposure to the air, carbohydrate content, and moisture level [15]. In our study, the *pH* of silage prepared from sugar-rich *SN* accessions varied from 4.57 to 5.61, with a mean value of 5.08 was more than 3.8–4.2, an ideal range for excellent quality silage [12] prepared from forages high in soluble sugar contents. Silage pH < 5.0 from IG-02-703, IG-2045-1, IG-02-713, and IG99-191 can be graded as good quality with lactic acid between 0.094 and 0.213 % DM. The lower lactic acid production in *SN* accessions possibly due to their adequate substrate (soluble sugar) for lactic acid bacteria. Our findings align with previous studies, where silages from tropical grasses *Pennisetum purpureum* and *Seteria splendid* had lower pH (3.96 and 4.07) and higher lactic acid production (2.53 and 2.47 % DM) compare to other studied tropical grasses, with value (4.71–5.32; and 1.04–1.84 % DM) [99,100]. *Paspalum plicatulum* grass silage pH & lactic acid were 5.2 &1.10 % DM and 5.2 & 1.80 % DM at 28 and 40 °C temperature for 30 days ensiling and 5.2 & 1.70 % DM and 5.1 & 2.0 % DM at 28 and 40 °C temperature for 60 days ensiling, respectively [101]. Silage from six warm season grasses exhibited low pH range 4–4.5 and high lactic acid production with range 3.91–7.65 % DM than our silage values [102]. *Pennisetum purpureum* silage had a higher pH (5.45) and lower lactic acid (0.90 % DM) than temperate ryegrass silage (3.86 and 1.90 % DM) [103]. Silage from five tropical grasses had pH values between 4.9 and 5.9, consistent with our findings [14].

## 5 Conclusions

The research underscored considerable genetic diversity within SN germplasm and sugar-rich accessions in terms of biomass yield, protein, fiber, energy, and sugar content. The sugar-rich accessions exhibited variations in carbohydrate and protein fractions, in vitro gas and methane production, and silage quality, including pH and lactic acid levels. Notably, accessions IG-02-703, IG-2045-1, IG-02-713, and IG99-191 showed promising ensiling potential, as the silage produced from these accessions had pH and lactic acid levels indicative of medium to good quality silage. Wide variability for methane to total gas production across sugar-rich accessions (0.107–0.178 across accessions to utilize the accessions for environment-friendly ruminant production. The present set of *Sehima* germplasm exhibits wide variability in biomass and forage quality traits, which hold significant potential. This variability can be be utilized to identify genomic regions and targeted biochemical pathways associated to important forage nutritional and ensiling traits. This will open new avenue for future breeding programs that utilize genomic selection to enhence forage biomass and superior nutritional quality in rangelands and pastures, thereby contributing to more sustainable livestock production.

## CRediT authorship contribution statement

**Sultan Singh:** Writing – review & editing, Writing – original draft, Methodology, Data curation, Conceptualization. **Tejveer Singh:** Writing – review & editing, Writing – original draft, Validation, Investigation, Formal analysis, Conceptualization. **Neeraj Kumar:** Writing – review & editing, Writing – original draft, Software, Formal analysis, Conceptualization. **Pushpendra Koli:** Writing – review & editing, Writing – original draft, Software, Methodology, Data curation. **Madan Mohan Das:** Writing – review & editing, Writing – original draft, Conceptualization. **Sanat Kumar Mahanta:** Writing – review & editing, Writing – original draft. **Krishna Kumar Singh:** Writing – review & editing, Writing – original draft. **Prakash K. Jha:** Writing – review & editing, Writing – original draft, Funding acquisition. **PV Vara Prasad:** Writing – review & editing, Writing – original draft, Funding acquisition. **Manoj Kumar Srivastava:** Writing – review & editing, Writing – original draft. **Rohit Katiyar:** Writing – review & editing, Writing – original draft.

## Data availability statement

Data will be made available on request.

## Funding

ICAR-Indian Grassland and Fodder Research under Institute Project No: CRSCIGFRICOL20130702 funded the present study.

## Declaration of competing interest

The authors declare that they have no known competing financial interests or personal relationships that could have appeared to influence the work reported in this paper.
