# Supplementary material for: Assessment of genetic diversity in Sehima nervosum for yield, nutritional traits and ensiling quality
Source: Heliyon. 2025 Jan 17;11(2):e42033. doi: 10.1016/j.heliyon.2025.e42033 (PMC11787658; doi:10.1016/j.heliyon.2025.e42033)
Supplement: Multimedia component 1 [file mmc1.doc]

Supplementary Table

Table S1. Nutritive value, sugar contents and yield of *Sehima nervosum* germplasm

| **Accession**  **identity** |  |  | | | | | | | | | | | | | | | | |
| --- | --- | --- | --- | --- | --- | --- | --- | --- | --- | --- | --- | --- | --- | --- | --- | --- | --- | --- |
|  | **CP** | **NDF** | **ADF** | **Cellulose** | **Lignin** | **Sugar** | **GFY I** | **GFY II** | **GFY T** | **DMI** | **DDM** | **RFV** | **TDN** | **DE** | **ME** | **NEM** | **NEL** | **NEG** |
| BS-1 | 4.5 | 72.7 | 48.1 | 33.5 | 6.1 | 85.8 | 48.4 | 6.1 | 54.5 | 1.6 | 51.5 | 64.2 | 42.4 | 1.9 | 1.6 | 1.2 | 0.9 | 0.2 |
| IG-2041-4 | 5.1 | 71.8 | 45.8 | 30.1 | 5.4 | 54.3 | 28.6 | 12.1 | 40.7 | 1.7 | 53.2 | 69.0 | 45.3 | 2.0 | 1.7 | 1.3 | 1.0 | 0.3 |
| IG95-92 | 4.5 | 73.0 | 50.6 | 31.0 | 5.4 | 41.7 | 47.5 | 8.2 | 55.7 | 1.6 | 49.5 | 63.0 | 39.0 | 1.8 | 1.4 | 1.1 | 0.8 | 0.1 |
| IG02-703 | 3.6 | 71.6 | 48.4 | 31.2 | 4.7 | 123.3 | 13.2 | 2.4 | 15.6 | 1.7 | 51.2 | 66.5 | 42.0 | 1.9 | 1.5 | 1.2 | 0.9 | 0.2 |
| IG02-706-1 | 4.7 | 74.9 | 53.0 | 33.5 | 5.5 | 37.8 | 13.2 | 7.9 | 21.1 | 1.6 | 47.6 | 59.1 | 35.9 | 1.6 | 1.3 | 1.0 | 0.8 | 0.0 |
| IG02-713-1 | 3.6 | 75.9 | 50.8 | 32.2 | 6.2 | 66.5 | 31.4 | 6.6 | 38.0 | 1.6 | 49.3 | 60.5 | 38.9 | 1.7 | 1.4 | 1.1 | 0.8 | 0.1 |
| IG2036 | 3.6 | 74.3 | 50.9 | 29.5 | 5.7 | 27.0 | 52.8 | 9.9 | 62.7 | 1.6 | 49.2 | 61.6 | 38.6 | 1.7 | 1.4 | 1.1 | 0.8 | 0.1 |
| IG-2044 | 4.0 | 77.3 | 48.4 | 30.9 | 5.4 | 56.6 | 12.0 | 9.4 | 21.4 | 1.6 | 51.2 | 61.6 | 42.0 | 1.9 | 1.5 | 1.2 | 0.9 | 0.2 |
| IG95-15 | 4.5 | 73.1 | 49.9 | 29.0 | 6.5 | 40.3 | 26.4 | 5.8 | 32.2 | 1.6 | 50.0 | 63.6 | 40.0 | 1.8 | 1.5 | 1.1 | 0.9 | 0.1 |
| IG95-185 | 4.7 | 68.8 | 48.4 | 28.9 | 3.9 | 39.5 | 22.6 | 9.4 | 32.1 | 1.7 | 51.2 | 69.2 | 41.9 | 1.9 | 1.5 | 1.2 | 0.9 | 0.2 |
| IG99-180 | 3.7 | 70.2 | 50.1 | 33.2 | 6.0 | 51.1 | 42.2 | 7.9 | 50.2 | 1.7 | 49.9 | 66.1 | 39.8 | 1.8 | 1.5 | 1.1 | 0.9 | 0.1 |
| IG01-349 | 4.3 | 71.8 | 46.0 | 30.4 | 5.5 | 93.4 | 23.8 | 6.6 | 30.4 | 1.7 | 53.1 | 68.8 | 45.1 | 2.0 | 1.7 | 1.3 | 1.0 | 0.3 |
| IG99-180A | 4.2 | 73.2 | 48.1 | 33.5 | 5.9 | 50.1 | 5.3 | 0.9 | 6.2 | 1.6 | 51.4 | 65.3 | 42.3 | 1.9 | 1.6 | 1.2 | 0.9 | 0.2 |
| IG99-198-1 | 4.3 | 71.4 | 51.8 | 28.6 | 5.7 | 67.7 | 26.4 | 5.9 | 32.3 | 1.7 | 48.6 | 63.3 | 37.6 | 1.7 | 1.4 | 1.1 | 0.8 | 0.1 |
| IG99-185 | 3.6 | 73.5 | 53.2 | 32.4 | 5.9 | 49.1 | 18.5 | 3.7 | 22.2 | 1.6 | 47.5 | 60.0 | 35.7 | 1.6 | 1.3 | 1.0 | 0.8 | 0.0 |
| IG95-185-1 | 4.2 | 74.5 | 50.0 | 34.9 | 6.3 | 62.3 | 29.0 | 10.1 | 39.2 | 1.6 | 49.9 | 62.3 | 39.8 | 1.8 | 1.5 | 1.1 | 0.9 | 0.1 |
| IG01-321-1 | 4.4 | 74.8 | 45.9 | 32.1 | 5.9 | 105.7 | 2.6 | 1.3 | 4.0 | 1.6 | 53.2 | 66.1 | 45.3 | 2.0 | 1.7 | 1.3 | 1.0 | 0.3 |
| IG99-188 | 3.6 | 79.5 | 50.2 | 33.9 | 5.8 | 75.2 | 10.6 | 2.6 | 13.2 | 1.5 | 49.8 | 58.3 | 39.6 | 1.8 | 1.5 | 1.1 | 0.8 | 0.1 |
| IG99-189 | 5.0 | 72.7 | 48.0 | 32.0 | 4.8 | 49.0 | 7.9 | 5.3 | 13.2 | 1.7 | 51.5 | 66.0 | 42.5 | 1.9 | 1.6 | 1.2 | 0.9 | 0.2 |
| IG99-193-1 | 5.0 | 76.8 | 54.3 | 32.1 | 6.6 | 61.2 | 21.1 | 6.6 | 27.7 | 1.6 | 46.6 | 56.4 | 34.3 | 1.5 | 1.3 | 1.0 | 0.7 | 0.0 |
| IG99-203 | 3.9 | 74.5 | 51.0 | 35.4 | 6.9 | 28.2 | 11.9 | 3.0 | 14.9 | 1.6 | 49.1 | 61.4 | 38.5 | 1.7 | 1.4 | 1.1 | 0.8 | 0.1 |
| IG99-195 | 5.3 | 75.1 | 48.5 | 32.5 | 6.2 | 82.9 | 31.7 | 11.2 | 42.8 | 1.6 | 51.1 | 63.5 | 41.8 | 1.9 | 1.5 | 1.2 | 0.9 | 0.2 |
| IG-2045-2 | 4.7 | 72.6 | 48.8 | 35.6 | 4.9 | 59.1 | 31.7 | 18.5 | 50.2 | 1.7 | 50.9 | 65.2 | 41.5 | 1.9 | 1.5 | 1.2 | 0.9 | 0.2 |
| IG2051-1 | 5.6 | 74.2 | 48.5 | 31.9 | 4.8 | 36.1 | 25.9 | 5.4 | 31.3 | 1.6 | 51.1 | 64.1 | 41.8 | 1.9 | 1.5 | 1.2 | 0.9 | 0.2 |
| IG-02-716 | 4.1 | 77.6 | 52.8 | 37.7 | 5.8 | 96.1 | 2.6 | 0.5 | 3.1 | 1.5 | 47.7 | 57.2 | 36.2 | 1.6 | 1.3 | 1.0 | 0.8 | 0.0 |
| IG-02-695 | 4.3 | 71.0 | 51.5 | 32.8 | 5.1 | 74.0 | 2.6 | 1.1 | 3.8 | 1.7 | 48.8 | 64.0 | 38.0 | 1.7 | 1.4 | 1.1 | 0.8 | 0.1 |
| IG02-712 | 5.0 | 70.5 | 48.7 | 30.6 | 5.0 | 73.5 | 25.6 | 4.4 | 30.0 | 1.7 | 51.0 | 67.3 | 41.6 | 1.9 | 1.5 | 1.2 | 0.9 | 0.2 |
| IG03-406-1 | 5.1 | 74.3 | 47.5 | 28.6 | 5.7 | 63.8 | 31.7 | 7.3 | 39.0 | 1.6 | 51.9 | 64.9 | 43.2 | 1.9 | 1.6 | 1.2 | 0.9 | 0.2 |
| IG03-406 | 4.6 | 72.8 | 50.9 | 33.2 | 5.9 | 79.3 | 33.0 | 9.9 | 42.9 | 1.6 | 49.2 | 62.9 | 38.7 | 1.7 | 1.4 | 1.1 | 0.8 | 0.1 |
| IG99-187 | 3.8 | 73.9 | 53.7 | 36.0 | 6.6 | 56.0 | 20.5 | 11.7 | 32.3 | 1.6 | 47.1 | 59.2 | 35.1 | 1.6 | 1.3 | 1.0 | 0.7 | 0.0 |
| IG99-204 | 3.7 | 72.3 | 49.3 | 32.7 | 5.3 | 47.9 | 29.3 | 11.7 | 41.1 | 1.7 | 50.5 | 65.0 | 40.8 | 1.8 | 1.5 | 1.2 | 0.9 | 0.2 |
| IG99-206 | 3.8 | 74.7 | 49.9 | 34.1 | 5.7 | 49.1 | 37.0 | 9.9 | 46.9 | 1.6 | 50.1 | 62.3 | 40.0 | 1.8 | 1.5 | 1.1 | 0.9 | 0.2 |
| IG-2041-1 | 5.0 | 74.2 | 48.8 | 32.1 | 5.0 | 57.8 | 19.8 | 8.8 | 28.6 | 1.6 | 50.9 | 63.7 | 41.4 | 1.9 | 1.5 | 1.2 | 0.9 | 0.2 |
| IG-2041-3 | 5.2 | 72.7 | 46.8 | 30.4 | 5.1 | 76.9 | 29.7 | 7.6 | 37.3 | 1.7 | 52.5 | 67.2 | 44.1 | 2.0 | 1.6 | 1.2 | 1.0 | 0.3 |
| IG-2045 | 4.1 | 75.0 | 48.9 | 30.3 | 4.4 | 58.2 | 38.5 | 9.4 | 47.9 | 1.6 | 50.8 | 63.0 | 41.3 | 1.9 | 1.5 | 1.2 | 0.9 | 0.2 |
| IG95-240 | 4.9 | 72.6 | 46.8 | 30.1 | 4.7 | 62.1 | 57.2 | 6.6 | 63.8 | 1.7 | 52.5 | 67.2 | 44.1 | 2.0 | 1.6 | 1.2 | 1.0 | 0.3 |
| IG95-52 | 4.0 | 73.6 | 48.6 | 30.1 | 6.0 | 59.8 | 24.0 | 4.8 | 28.8 | 1.6 | 51.0 | 64.5 | 41.7 | 1.9 | 1.5 | 1.2 | 0.9 | 0.2 |
| IG99-179 | 5.1 | 75.4 | 44.3 | 25.0 | 4.5 | 48.0 | 23.8 | 9.0 | 32.7 | 1.6 | 54.4 | 67.1 | 47.3 | 2.1 | 1.7 | 1.3 | 1.0 | 0.4 |
| IG99-181-2 | 4.1 | 70.2 | 44.4 | 29.5 | 6.0 | 77.6 | 26.4 | 3.8 | 30.2 | 1.7 | 54.3 | 72.0 | 47.2 | 2.1 | 1.7 | 1.3 | 1.0 | 0.4 |
| IG-02-695-1 | 3.7 | 75.3 | 45.0 | 29.3 | 5.5 | 102.0 | 25.9 | 8.3 | 34.1 | 1.6 | 53.9 | 66.5 | 46.4 | 2.1 | 1.7 | 1.3 | 1.0 | 0.3 |
| IG99-181-1 | 3.3 | 72.6 | 47.9 | 32.0 | 5.0 | 62.6 | 20.5 | 4.4 | 24.9 | 1.7 | 51.6 | 66.1 | 42.7 | 1.9 | 1.6 | 1.2 | 0.9 | 0.2 |
| IG99-183 | 5.3 | 75.6 | 47.8 | 26.6 | 6.1 | 45.5 | 39.6 | 2.2 | 41.8 | 1.6 | 51.7 | 63.5 | 42.7 | 1.9 | 1.6 | 1.2 | 0.9 | 0.2 |
| IG99-192 | 3.7 | 78.2 | 53.5 | 32.2 | 5.2 | 36.9 | 52.8 | 9.9 | 62.7 | 1.5 | 47.2 | 56.1 | 35.3 | 1.6 | 1.3 | 1.0 | 0.7 | 0.0 |
| IG99-194 | 3.2 | 76.3 | 51.9 | 35.1 | 6.8 | 45.6 | 7.9 | 10.6 | 18.5 | 1.6 | 48.5 | 59.1 | 37.5 | 1.7 | 1.4 | 1.1 | 0.8 | 0.1 |
| IG99-195-1 | 4.5 | 73.5 | 49.1 | 32.3 | 5.0 | 60.7 | 26.4 | 12.8 | 39.2 | 1.6 | 50.7 | 64.1 | 41.1 | 1.8 | 1.5 | 1.2 | 0.9 | 0.2 |
| IG99-197 | 4.7 | 72.4 | 47.8 | 34.8 | 7.5 | 47.8 | 44.0 | 5.5 | 49.5 | 1.7 | 51.6 | 66.3 | 42.7 | 1.9 | 1.6 | 1.2 | 0.9 | 0.2 |
| IG99-197-1 | 4.1 | 73.3 | 48.7 | 32.4 | 6.1 | 66.1 | 47.1 | 5.8 | 52.9 | 1.6 | 51.0 | 64.7 | 41.6 | 1.9 | 1.5 | 1.2 | 0.9 | 0.2 |
| IG99-199 | 4.1 | 78.9 | 49.7 | 33.2 | 6.1 | 63.4 | 46.5 | 11.9 | 58.3 | 1.5 | 50.2 | 59.2 | 40.3 | 1.8 | 1.5 | 1.1 | 0.9 | 0.2 |
| IG02-669 | 4.0 | 73.3 | 50.1 | 29.9 | 4.9 | 75.0 | 2.6 | 10.0 | 12.7 | 1.6 | 49.8 | 63.2 | 39.7 | 1.8 | 1.5 | 1.1 | 0.9 | 0.1 |
| IG99-201 | 4.8 | 71.3 | 46.1 | 30.3 | 5.6 | 72.5 | 55.4 | 6.1 | 61.5 | 1.7 | 53.0 | 69.1 | 44.9 | 2.0 | 1.7 | 1.3 | 1.0 | 0.3 |
| IG99-202 | 3.2 | 73.8 | 51.8 | 35.6 | 5.7 | 42.7 | 33.0 | 9.9 | 42.9 | 1.6 | 48.5 | 61.2 | 37.5 | 1.7 | 1.4 | 1.1 | 0.8 | 0.1 |
| IG-99-198 | 3.6 | 74.2 | 46.9 | 31.3 | 6.1 | 88.8 | 29.0 | 9.5 | 38.5 | 1.6 | 52.4 | 65.6 | 43.9 | 2.0 | 1.6 | 1.2 | 1.0 | 0.3 |
| IG02-717 | 4.1 | 75.3 | 49.2 | 30.8 | 5.6 | 63.0 | 58.1 | 25.9 | 84.0 | 1.6 | 50.5 | 62.4 | 40.9 | 1.8 | 1.5 | 1.2 | 0.9 | 0.2 |
| IG95-57 | 3.7 | 77.0 | 51.0 | 34.2 | 5.1 | 72.0 | 35.8 | 17.7 | 53.5 | 1.6 | 49.1 | 59.3 | 38.5 | 1.7 | 1.4 | 1.1 | 0.8 | 0.1 |
| IG95-61 | 4.3 | 77.7 | 50.8 | 31.8 | 6.8 | 41.0 | 51.3 | 20.9 | 72.2 | 1.5 | 49.3 | 59.1 | 38.8 | 1.7 | 1.4 | 1.1 | 0.8 | 0.1 |
| IG-2051 | 5.0 | 77.0 | 45.4 | 32.5 | 6.0 | 34.0 | 13.2 | 7.6 | 20.8 | 1.6 | 53.6 | 64.7 | 45.9 | 2.1 | 1.7 | 1.3 | 1.0 | 0.3 |
| IG01-307-3 | 4.3 | 77.7 | 48.1 | 33.1 | 7.5 | 62.8 | 39.6 | 13.2 | 52.8 | 1.5 | 51.1 | 61.2 | 41.8 | 1.9 | 1.5 | 1.2 | 0.9 | 0.2 |
| IG01-349-1 | 4.9 | 72.8 | 47.8 | 30.9 | 5.9 | 70.8 | 47.0 | 17.6 | 64.6 | 1.6 | 51.7 | 66.1 | 42.8 | 1.9 | 1.6 | 1.2 | 0.9 | 0.2 |
| IG99-199-1 | 4.8 | 72.9 | 47.3 | 29.4 | 6.3 | 50.7 | 49.5 | 26.4 | 75.9 | 1.6 | 52.0 | 66.4 | 43.4 | 1.9 | 1.6 | 1.2 | 0.9 | 0.2 |
| IG01-307-1 | 3.9 | 75.7 | 51.7 | 33.5 | 6.3 | 45.4 | 44.0 | 30.8 | 74.8 | 1.6 | 48.6 | 59.7 | 37.7 | 1.7 | 1.4 | 1.1 | 0.8 | 0.1 |
| IG01-314 | 5.1 | 71.9 | 47.8 | 29.1 | 6.0 | 27.6 | 26.4 | 3.8 | 30.2 | 1.7 | 51.7 | 66.9 | 42.8 | 1.9 | 1.6 | 1.2 | 0.9 | 0.2 |
| IG01-329-1 | 3.6 | 77.7 | 51.1 | 31.9 | 5.4 | 60.0 | 56.2 | 11.7 | 67.9 | 1.5 | 49.1 | 58.8 | 38.4 | 1.7 | 1.4 | 1.1 | 0.8 | 0.1 |
| IG01-349-2 | 4.6 | 71.1 | 51.9 | 31.9 | 6.4 | 66.9 | 42.2 | 11.9 | 54.1 | 1.7 | 48.5 | 63.4 | 37.4 | 1.7 | 1.4 | 1.1 | 0.8 | 0.1 |
| IG99-193-2 | 4.4 | 72.6 | 43.7 | 30.4 | 4.5 | 66.4 | 38.3 | 7.9 | 46.2 | 1.7 | 54.9 | 70.3 | 48.1 | 2.2 | 1.8 | 1.4 | 1.1 | 0.4 |
| IG01-338 | 4.1 | 76.5 | 48.8 | 31.6 | 5.3 | 46.7 | 38.3 | 6.5 | 44.8 | 1.6 | 50.9 | 61.9 | 41.4 | 1.9 | 1.5 | 1.2 | 0.9 | 0.2 |
| IG95-315 | 3.4 | 70.7 | 47.5 | 30.9 | 5.0 | 82.4 | 23.5 | 5.3 | 28.8 | 1.7 | 51.9 | 68.3 | 43.1 | 1.9 | 1.6 | 1.2 | 0.9 | 0.2 |
| IG2000-72 | 5.1 | 80.1 | 52.0 | 34.4 | 5.9 | 52.0 | 16.5 | 23.1 | 39.6 | 1.5 | 48.4 | 56.2 | 37.3 | 1.7 | 1.4 | 1.1 | 0.8 | 0.1 |
| IG-99-186 | 4.5 | 77.4 | 48.5 | 34.1 | 5.5 | 81.9 | 37.0 | 18.5 | 55.4 | 1.5 | 51.1 | 61.4 | 41.8 | 1.9 | 1.5 | 1.2 | 0.9 | 0.2 |
| IG01-307-2 | 4.9 | 73.0 | 47.8 | 30.3 | 4.9 | 43.8 | 31.7 | 5.3 | 37.0 | 1.6 | 51.7 | 65.9 | 42.7 | 1.9 | 1.6 | 1.2 | 0.9 | 0.2 |
| IG01-391-1 | 4.8 | 73.6 | 48.5 | 32.4 | 5.2 | 58.9 | 29.0 | 6.6 | 35.6 | 1.6 | 51.1 | 64.6 | 41.8 | 1.9 | 1.5 | 1.2 | 0.9 | 0.2 |
| IG02-699-1 | 3.4 | 72.5 | 49.7 | 35.5 | 5.0 | 65.0 | 33.0 | 11.2 | 44.2 | 1.7 | 50.2 | 64.3 | 40.3 | 1.8 | 1.5 | 1.1 | 0.9 | 0.2 |
| IG02-699 | 3.8 | 72.7 | 45.6 | 31.2 | 4.6 | 60.6 | 39.6 | 10.6 | 50.2 | 1.7 | 53.4 | 68.3 | 45.6 | 2.0 | 1.7 | 1.3 | 1.0 | 0.3 |
| IG02-699-2 | 5.3 | 70.6 | 46.1 | 28.1 | 5.7 | 53.3 | 23.5 | 10.0 | 33.5 | 1.7 | 53.0 | 69.8 | 45.0 | 2.0 | 1.7 | 1.3 | 1.0 | 0.3 |
| IG01-319 | 6.0 | 74.6 | 46.7 | 30.2 | 5.7 | 71.1 | 17.7 | 5.5 | 23.2 | 1.6 | 52.5 | 65.5 | 44.2 | 2.0 | 1.6 | 1.3 | 1.0 | 0.3 |
| IG-2041-2 | 5.3 | 71.1 | 47.6 | 33.9 | 4.3 | 84.4 | 23.0 | 10.3 | 33.2 | 1.7 | 51.9 | 67.8 | 43.1 | 1.9 | 1.6 | 1.2 | 0.9 | 0.2 |
| IG-2045-1 | 3.7 | 77.0 | 47.5 | 33.2 | 4.9 | 115.8 | 26.1 | 13.2 | 39.3 | 1.6 | 51.9 | 62.7 | 43.1 | 1.9 | 1.6 | 1.2 | 0.9 | 0.2 |
| IG02-702 | 4.7 | 71.5 | 50.0 | 27.2 | 5.2 | 61.0 | 18.5 | 7.9 | 26.4 | 1.7 | 49.9 | 64.9 | 39.8 | 1.8 | 1.5 | 1.1 | 0.9 | 0.1 |
| IG02-702A | 3.1 | 70.6 | 52.2 | 31.6 | 5.6 | 57.7 | 18.5 | 10.6 | 29.0 | 1.7 | 48.2 | 63.5 | 37.0 | 1.7 | 1.4 | 1.0 | 0.8 | 0.1 |
| IG99-193 | 4.2 | 69.2 | 48.2 | 27.7 | 5.4 | 76.7 | 19.8 | 9.9 | 29.7 | 1.7 | 51.4 | 69.0 | 42.2 | 1.9 | 1.6 | 1.2 | 0.9 | 0.2 |
| IG01-307 | 3.2 | 70.7 | 47.6 | 30.0 | 4.8 | 85.9 | 23.0 | 11.7 | 34.7 | 1.7 | 51.8 | 68.1 | 42.9 | 1.9 | 1.6 | 1.2 | 0.9 | 0.2 |
| IG99-181 | 3.5 | 70.8 | 50.5 | 28.8 | 4.1 | 84.0 | 5.3 | 2.3 | 7.5 | 1.7 | 49.5 | 65.1 | 39.2 | 1.8 | 1.4 | 1.1 | 0.8 | 0.1 |
| IG02-703-1 | 4.6 | 71.0 | 48.7 | 26.6 | 4.6 | 66.1 | 5.3 | 2.6 | 7.9 | 1.7 | 51.0 | 66.8 | 41.6 | 1.9 | 1.5 | 1.2 | 0.9 | 0.2 |
| IG-02-706 | 3.6 | 72.5 | 49.2 | 30.5 | 4.7 | 85.4 | 2.6 | 2.0 | 4.6 | 1.7 | 50.5 | 64.9 | 40.9 | 1.8 | 1.5 | 1.2 | 0.9 | 0.2 |
| IG02-715 | 4.4 | 76.7 | 50.3 | 33.4 | 7.9 | 68.8 | 19.8 | 2.0 | 21.8 | 1.6 | 49.7 | 60.3 | 39.5 | 1.8 | 1.5 | 1.1 | 0.8 | 0.1 |
| IG01-391 | 3.8 | 75.0 | 47.6 | 32.1 | 6.6 | 87.2 | 25.9 | 10.6 | 36.4 | 1.6 | 51.8 | 64.2 | 42.9 | 1.9 | 1.6 | 1.2 | 0.9 | 0.2 |
| IG02-713 | 3.1 | 72.1 | 46.8 | 31.0 | 6.9 | 90.1 | 26.1 | 11.9 | 38.0 | 1.7 | 52.4 | 67.7 | 44.0 | 2.0 | 1.6 | 1.2 | 1.0 | 0.3 |
| IG02-704 | 3.6 | 76.6 | 51.9 | 33.0 | 7.2 | 47.8 | 2.2 | 1.6 | 3.8 | 1.6 | 48.5 | 58.9 | 37.4 | 1.7 | 1.4 | 1.1 | 0.8 | 0.1 |
| IG02-705A | 4.6 | 72.9 | 49.4 | 29.7 | 5.0 | 45.8 | 13.2 | 7.9 | 21.1 | 1.6 | 50.4 | 64.3 | 40.7 | 1.8 | 1.5 | 1.2 | 0.9 | 0.2 |
| IG02-709 | 4.4 | 74.4 | 50.1 | 29.2 | 6.8 | 40.4 | 33.0 | 6.3 | 39.3 | 1.6 | 49.8 | 62.3 | 39.7 | 1.8 | 1.5 | 1.1 | 0.9 | 0.1 |
| IG99-191 | 3.9 | 73.3 | 45.7 | 28.7 | 5.3 | 83.1 | 15.8 | 9.0 | 24.8 | 1.6 | 53.3 | 67.7 | 45.5 | 2.0 | 1.7 | 1.3 | 1.0 | 0.3 |
| IG99-178 | 4.5 | 73.8 | 48.9 | 31.6 | 5.7 | 63.3 | 55.4 | 26.4 | 81.8 | 1.5 | 54.9 | 66.3 | 39.8 | 1.2 | 1.4 | 1.2 | 0.9 | 0.2 |
| IG-2044-1 | 4.3 | 73.8 | 49.0 | 31.6 | 5.6 | 63.3 | 29.6 | 11.9 | 41.5 | 1.6 | 55.0 | 64.5 | 38.8 | 1.3 | 1.5 | 1.1 | 0.9 | 0.2 |

CP, Crude protein % DM; NDF, Neutral detergent fibre % DM; ADF, Acid detergent fibre % DM; Cellulose % DM; Lignin, % DM; Sugar, mg g^-1^ DM; GFY I, Green fodder yield t ha^-1^ (rainy season), GFY II, Green fodder yield t ha^-1^ (summer season); GFY T, Green fodder yield t ha^-1^ (total); DMI, Dry matter intake %; DDM, Digestible dry mater % DM; RFV, Relative feed value %; TDN, Total digestible nutrients % DM; DE, Digestible energy Mcal kg^-1^ DM; ME, Metabolizable energy Mcal kg^-1^ DM; NE_M_, Net energy for maintenance Mcal kg^-1^ DM; NE_L_, Net energy for lactation Mcal kg^-1^ DM; NE_G_, Net energy for growth/gain Mcal kg^-1^ DM.

Table S2. Broad sense Heritability of forage quality traits of *Sehima nervosum* germplasm

| **Quality trait** | **Heritability** |
| --- | --- |
| NDF (%) | 0.69 |
| ADF (%) | 0.76 |
| Cellulose | 0.64 |
| Lignin | 0.41 |
| CP (%) | 0.87 |
| N2(%) | 0.92 |
| Sugar | 0.98 |
| Starch | 0.93 |

Table S3. Distribution of *Sehima nervosum* genotypes by geographic area

| **Area/State** | **Genotypes** |
| --- | --- |
| Western Ghats | IG03-402 to IG03-406, IG96-71-74, IG96-169 to IG96-174, IG96-185, IG96-245, IG96-258, IG96-263 to 267, IG96-273, IG96-290 to 292 |
| Maharashtra | IG02-699 to IG02-718, IG01-276, IG01-294, IG01-304, IG01-307, IG01-314, IG01-321, IG01-329, IG01-331 to IG01-333, IG01-338, IG01-349, IG01-353, IG01-370, IG01-378, IG01-391, IG01-394, IG01-397 to IG01-398, IG01-414, IG01-420, IG01-424 |
| Madhya Pradesh | IG01-511 to IG01-512, IG2000-75, IG2000-91, IG95-210 to 227, IG95-235 to 269, IG95-325 to 328, IG95-382 to 396, IG95-438 to 439 |
| Uttar Pradesh | IG2000-72, IG97161-162, IG97-254 to 256, IG96-300 to 304, IG9503-21, IG95-40 to IG95-92, IG95-125, IG95-160 to 168 |
| Southern Rajasthan | IG99-176 to IG99-206 |
| Rajasthan | IG97-573 to 599 |
| Karnataka | IG93-3177 to 3185, IG93-3193, IG93-3196 to 3197, IG93-3201 |
